# Designing the Future of Biosensing: Advances in Aptamer Discovery, Computational Modeling, and Diagnostic Applications

**DOI:** 10.3390/bios15100637

**Published:** 2025-09-24

**Authors:** Robert G. Jesky, Louisa H. Y. Lo, Ryan H. P. Siu, Julian A. Tanner

**Affiliations:** 1 School of Biomedical Sciences, Faculty of Medicine, The Chinese University of Hong Kong, Hong Kong SAR, China; robertjesky@cuhk.edu.hk; 2 Advanced Biomedical Instrumentation Centre, Hong Kong Science and Technology Park (HKSTP), Hong Kong SAR, China; louisa.lo@abic.hk; 3 School of Biomedical Sciences, LKS Faculty of Medicine, The University of Hong Kong, Hong Kong SAR, China; u3008168@connect.hku.hk; 4 Material Innovation Institute for Life Sciences and Energy (MILES), HKU-Shenzhen Institute of Research and Innovation (HKU-SIRI), Shenzhen 518057, China; 5 School of Biomedical Engineering, The University of Hong Kong, Hong Kong SAR, China

**Keywords:** aptamer, SELEX, artificial intelligence, machine learning, biosensor, point-of-care diagnostic

## Abstract

Recent advances in computational tools, particularly machine learning (ML), deep learning (DL), and structure-based modeling, are transforming aptamer research by accelerating discovery and enhancing biosensor development. This review synthesizes progress in predictive algorithms that model aptamer–target interactions, guide in silico sequence optimization, and streamline design workflows for both laboratory and point-of-care diagnostic platforms. We examine how these approaches improve key aspects of aptasensor development, such as aptamer selection, sensing surface immobilization, signal transduction, and molecular architecture, which contribute to greater sensitivity, specificity, and real-time diagnostic capabilities. Particular attention is given to illuminating the technological and experimental advances in structure-switching aptamers, dual-aptamer systems, and applications in electrochemical, optical, and lateral flow platforms. We also discuss current challenges such as the standardization of datasets and interpretability of ML models and highlight future directions that will support the translation of aptamer-based biosensors into scalable, point-of-care and clinically deployable diagnostic solutions.

## 1. Introduction

Aptamers are short, single-stranded oligonucleotides which exhibit a marked capacity to bind to a broad spectrum of molecular targets with high specificity and affinity. They have thus emerged as effective alternatives to antibodies in diagnostics and biosensing. Additional characteristics such as their chemical stability, structural malleability, and synthetic accessibility make them particularly appealing for incorporation into next-generation biosensors. Over the past couple of decades, advances in aptamer research along with breakthroughs in molecular selection techniques and computational biology have led to a convergence of experimental approaches, leading to significant improvements in sensitivity, selectivity, and scalability.

Traditional methods of aptamer selection via Systematic Evolution of Ligands by Exponential Enrichment (SELEX), while effective, often suffer from long iteration cycles and limited success against complex targets. Recent innovations in magnetic bead-based SELEX, capture SELEX, and capillary electrophoresis SELEX have streamlined critical aspects of the selection process. Likewise, conventional screening methods are being augmented and/or replaced by harnessing innovations in machine learning (ML) that are in turn leading to accelerated identification and optimization of functional aptamer sequences. A host of computational tools that range from structure-based modeling and docking simulations to predictive algorithms and generative Artificial intelligence (AI) frameworks are reshaping the processes of aptamer discovery and enabling high-throughput, in silico design strategies.

Bridging bioengineering with computational design has led to a wide range of aptamer-based biosensing platforms, including lateral flow assays (LFAs), electrochemical, optical, and even thermal modalities. Each one offers unique advantages in terms of diagnostic performance, speed and portability. Broader integration of nanomaterials along with advances in immobilization strategies, and signal amplification have further elevated aptasensors’ analytical performance. Moreover, structure-switching aptamers, dual-aptamer systems, and deep learning (DL)-based pipelines illustrate how fine-tuned molecular design can enhance the responsiveness and multiplexing capabilities of biosensors. Such technological advancements are driving laboratory innovation and expanding the versatility necessary for tangible application.

At the forefront of research sits the integration of experimental and computational techniques that together are driving a pivotal shift in the development and application of aptasensors. This paper provides a detailed overview of the significant progress that has been made in biosensor design, aptamer optimization, computational modeling and point-of-care implementation. Application of combinatorial methods is accelerating the evolution of high-performance, next-generation aptasensors that offer broader application in diagnostics and personalized medicine. While earlier reviews have categorized aptasensor development according to biosensor types [1,2,3,4], this work provides a distinct perspective by critically linking state-of-the-art computational models for aptamer optimization to innovations in biosensor engineering and real-world diagnostics. We offer a cross-dimensional and comprehensive analysis that goes beyond prior reviews and ensures relevance to the rapidly evolving intersection of AI and clinical biosensing. The main purpose of this review is to provide a thorough, forward-looking account of recent progress with notable focus on the translation of aptasensor innovations into clinical and commercial solutions. Having synthesized notable advances in the field, we also aim to have this work serve as a foundational resource that supports ongoing research and helps orient future efforts toward scalable, real-world applications.

## 2. Advances in Aptamer Selection and Computational Approaches

### 2.1. Overview of Aptamer Selection Strategies

Aptamers are short, single-stranded oligonucleotides capable of folding into distinct secondary and tertiary structures, allowing them to bind a wide range of targets including proteins [5], small molecules [6,7], and whole cells [8], with high affinity and specificity. Aptamers can be synthesized chemically without the use of animals, offering a consistent and cost-effective alternative to antibody production and usage [9,10]. The CRISPR/Cas system is another recognition element that has attracted growing interest for its programmable nucleic acid-based recognition with high specificity. However, its design is mainly limited to nucleic acid targets and often requires auxiliary enzymes for detection [11,12]. Molecularly imprinted polymers (MIP), in contrast, are synthetic materials with robustness and stability under harsh conditions, though they often experience limited binding site homogeneity and lower affinity compared to aptamers [13,14]. Collectively, the application of aptamers has gained increasing attention in terms of their use in therapeutics [15] and biosensor development, especially in point-of-care diagnostics and screening, due to their comparative advantages of thermal stability, high sensitivity and flexibility of modification [2,16]. Therefore, efficient methods of aptamer selection have become fundamental in supporting the advancement of biosensor invention.

SELEX remains the elemental technique for generating aptamers [17,18]. The classical SELEX workflow starts with a large pool of random and unique sequences of oligonucleotides (the “library”), which involves iterative rounds of target binding and partitioning of bound from unbound sequences. The bound pool is amplified (e.g., PCR for DNA aptamers, or RT-PCR for RNA aptamers) in each round to progressively enrich for high-affinity candidates. Over time, various aptamer selection strategies have been introduced to expand its applicability and efficiency towards different types of targets [19,20]. Some of the common methods are discussed in this context.

#### 2.1.1. Magnetic Bead-Based SELEX

**Figure 1:**
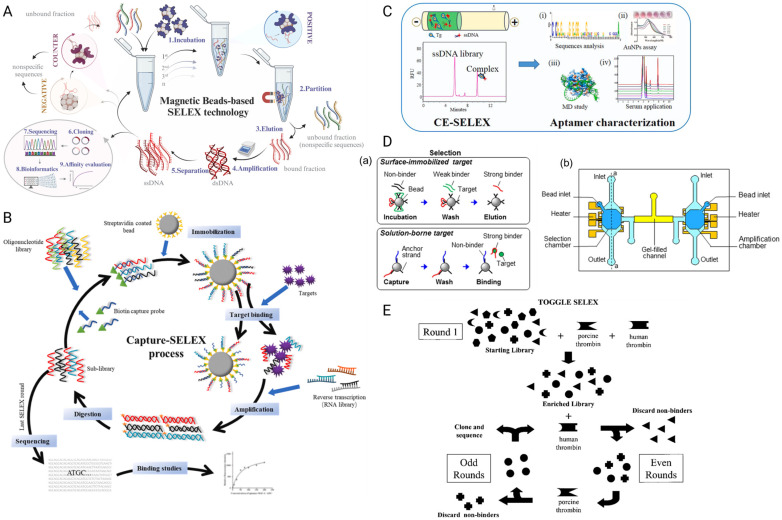
Representative designs of various SELEX strategies are illustrated here. (**A**) Magnetic-bead based SELEX. The target is immobilized on magnetic beads, enabling straightforward separation of bound sequences from the unbound pool by applying a magnetic field. Bound sequences are PCR-amplified, and the selection is iterated until enrichment is achieved. Figure modified from [24]. Copyright © 2024 Elsevier B.V. All rights reserved. (**B**) Capture SELEX. This strategy starts by immobilizing the library via a complementary capture strand. The targets are introduced in solution to induce target-dependent strand displacement. The released target-binding sequences are PCR-amplified, and the selection is repeated until enrichment is achieved. Figure modified from [28]. Copyright © 2021 Elsevier B.V. All rights reserved. (**C**) Capillary electrophoresis SELEX. By applying an electric field, complexes containing the bound sequences are separated from free oligonucleotides based on their electrophoretic mobility shifts. This method allows rapid and efficient selection and the selected aptamers can be characterized by various downstream studies. (i) Sequence homology analysis using Clustal X software to identify the conserved bases that possibly participate in target binding. (ii) The affinity of the selected aptamer was confirmed qualitatively by gold nanoparticles colorimetric assay. (iii) Molecular dynamics simulation of the aptamer-target binding. (iv) Aptamer-based target detection in spiked serum matrix, indicating the potential of real application. [33]. Copyright © 2020 Elsevier B.V. All rights reserved. (**D**) Microchip-mediated selection and amplification of Microfluidic SELEX. (**a**) Oligonucleotides are selected on microbeads against surface-immobilized or solution-phase target. Bound sequences are then collected and amplified. (**b**) This principle can be implemented in a microchip consisting of the selection and amplification microchambers. Figure modified from [37], licensed under CC BY 4.0. (**E**) Toggle-SELEX. Selection pressure is alternated across different but related targets, enriching for aptamers that recognize conserved structural features with broader applicability. Figure reproduced from [39], licensed under CC BY 4.0.

This is one of the widely used strategies in selecting aptamers through binding with the target biomolecules with known tags or surface characteristics. The tagged targets are conjugated onto magnetic beads via linkers, for example, Ni-NTA coupling (for His-tagged targets), streptavidin coupling (for biotinylated targets) or other covalent coupling [21,22,23]. The oligonucleotide library is incubated with the bead-target complexes to allow interaction with exposed binding sites. A magnetic field is then applied to separate the beads (carrying the bound oligonucleotides) from the supernatant (carrying the unbound population). Following the appropriate washing steps to remove the non-specific binders, the bound sequences are eluted and amplified. As shown in Figure 1A, the approach is efficient and fast due to the simplified partitioning step, and the selectivity can be easily adjusted by simply manipulating the amount of bead-target complexes used and introducing the additional negative/counter selection steps [24]. However, this approach is often limited by the availability of the exposed target binding sites required for aptamer interaction after target conjugation with the magnetic beads. Therefore, another modified strategy called Capture SELEX is introduced.

#### 2.1.2. Capture SELEX

Figure 1B illustrates the core steps of capture SELEX (such as incubation, elution and amplification) that are similar to the magnetic beads-based SELEX, except that it represents a reverse approach, where the oligonucleotide library is immobilized on the solid support instead of the target biomolecules. Typically, the oligonucleotide candidates are biotinylated and conjugated via the biotin-streptavidin interaction on the streptavidin coated solid support [25,26]. The target biomolecules are then passed through the library and the bound oligonucleotides are “captured”, which are released into the supernatant and collected for further purification and amplification [27]. This approach is especially useful for aptamer selection against small biomolecules with limited immobilization surface and binding epitopes [28]. By allowing the target biomolecules to retain their native conformation, it is also reported that the approach allows selection of aptamers with structure-switching properties upon target binding [29,30], suggesting it may be advantageous for downstream applications.

#### 2.1.3. Capillary Electrophoresis SELEX (CE-SELEX)

In this approach, the oligonucleotide library is first incubated with the targets. As the binding results in a measurable change in molecular size or charge, the aptamer-target complexes migrate differently under an electric field (Figure 1C). Due to the difference in electrophoretic mobility between the complexes and the unbound oligonucleotides, the bound sequences can be separated efficiently via capillary electrophoresis [31]. Affinity maturation by CE-SELEX is highly efficient, and it is reported that the whole selection procedure can be completed in as little as 2–4 rounds [32,33].

#### 2.1.4. Microfluidic SELEX

A more recent approach involves microscale fluid handling systems that automate the aptamer selection process (Figure 1D). By miniaturizing the separation interface (such as on-chip), these systems dramatically increase the surface-to-volume ratio, which enhances the efficiency of target-binding separation and reduces reagent consumption [34]. These advantages over classical SELEX have facilitated the development of diverse microfluidic SELEX variations, including bead-based, sol–gel, and electrophoresis-based formats [35,36,37], as well as emerging approaches like Pro-SELEX, which integrates microfluidic sorting with bioinformatics to isolate aptamers with programmable binding affinities in a single selection round [38].

#### 2.1.5. Alternative Aptamer Selection Strategies

In addition to conventional SELEX and its common variants, alternative aptamer selection strategies have been proposed to improve selection versatility, reduce screening time and broaden target specificity. These include multi-target cycling methods and approaches that integrate computational prediction to streamline candidate enrichment. Toggle-SELEX, a modified technique introduced in 2001, allows for the screening of cross-reactive or broad-spectrum aptamers by alternating multiple related targets between selection rounds (Figure 1E) [39]. Similarly, in silico-enhanced SELEX leverages computational methods such as library pre-screening and structural modeling to improve candidate identification efficiency [40,41]. These approaches are often considered cost-effective, as they require fewer laboratory resources, reduce the number of wet-lab selection rounds, and significantly accelerate aptamer discovery [42].

In summary, ongoing innovations in aptamer selection methods illustrate the complexity and sophistication of experimental procedures required to improve selection efficiency and adaptability. However, despite these methodological advances, the SELEX process still faces several limitations that hinder its broader applicability, especially in high-throughput or precision-driven contexts. These challenges are discussed in the next section.

### 2.2. Challenges in Conventional SELEX

Despite the vital role of SELEX in aptamer discovery, several challenges persist, particularly limitations in efficiency and success rate. Optimization strategies are often discussed in an attempt to improve and monitor the outcome of the aptamer selection [43,44].

One of the most common drawbacks is the time-consuming and labor-intensive nature of the process [45]. A typical SELEX workflow requires multiple rounds (usually 8–15 rounds) of iterative target binding, oligonucleotide amplification, and separation. This process often spans several weeks and demands careful optimization and post-selection characterization for each specific target.

Another pitfall is the uncertainty of success [46]. As the amplification proceeds, it may introduce non-specific enrichment by favoring sequences with higher amplification efficiency, despite their binding affinity is suboptimal [47]. These unwanted by-products may dominate the final pool due to PCR amplification bias [48]. Furthermore, the diversity of potential targets introduces structural complexity and variability in accessibility, which can complicate the aptamer binding [49]. To improve the target binding specificity, sufficient counter-selection strategies are often included between positive selection rounds to eliminate false-positive binding sequences via general charge attraction or hydrophobic interactions [50,51].

Additional concern lies in the reproducibility of aptamer selection outcomes. Targets expressed in prokaryotic cells, such as recombinant proteins or complexes, are often used instead of those expressed in eukaryotic cells due to purification challenges. This could, however, create binding performance discrepancies in actual applications due to the presence of post-translational modifications [52]. Moreover, other laboratory factors, such as the diversity of library design, the choice of selection buffers, and the selection stringency, can influence the enrichment outcomes even with the same target [53].

Taken together, these challenges highlight the need for more rational and controlled experimental planning to minimize variation and bias throughout the selection process. Beyond improving the SELEX design, several post-SELEX optimization strategies have also been developed to enhance aptamer performance. These include truncation, chemical modification, multivalency, and mutagenesis, each aim to improve properties such as binding affinity, nuclease resistance, or thermal stability for targeted applications [54].

In parallel, computational and AI-based approaches are gaining traction as transformative tools to accelerate aptamer discovery through in silico modeling and rational design.

### 2.3. AI-Driven Strategies for Aptamer Discovery and Design

AI and ML techniques are gaining considerable adoption in the field of aptamer discovery, due to their excellent data-driven computational capacity to learn from patterns, especially from large-scale SELEX datasets, and their ability to predict the desirable aptamer sequences with preferable binding properties across diverse targets [55].

#### 2.3.1. Pattern Recognition

One of the core applications of AI in aptamer research lies in pattern recognition, where ML models are used to classify or score aptamer sequences based on features correlated with binding performance, an approach that contrasts with the experimental trial-and-error of classical SELEX.

For instance, utilizing structural and topological descriptors as input features, support vector machine (SVM) models have been used to distinguish high- and low-affinity aptamers targeting streptavidin [56]. In a related study focused on hepatocellular carcinoma (SMMC-7721) cells, a similar structure-activity relationship approach was used, in which over 1600 molecular descriptors were extracted from cell-SELEX derived sequences and used to train SVMs optimized via particle swarm algorithms. Six aptamer sequences were experimentally validated with nanomolar binding affinities [57]. Both models achieved high prediction accuracy, and their outputs were consistent with the evolutionary principles of SELEX, supporting the use of SVMs as effective tools for identifying high-affinity aptamer candidates.

To expand upon this, a sequence-based pattern recognition strategy has also been demonstrated. A Natural Language Processing (NLP) technique known as CountVectorizer was used to convert nucleotide sequences into numerical representations of 6-mer frequencies. These features were analyzed using several classifiers, including an SVM, which achieved exceptional performance (AUROC = 0.998) in distinguishing functional aptamers from genomic DNA backgrounds [58]. This highlights the adaptability of pattern recognition across different sequence encoding methods.

Beyond SVMs, other classifiers have also been investigated. By combining features from the aptamer sequence (nucleotide composition) and the target protein (amino acid and pseudo-amino acid composition), one ML approach used a Random Forest (RF) model to predict aptamer-target interactions [59]. With optimized feature selection using the maximum relevance minimum redundancy (mRMR) method and incremental feature selection, the final model achieved 77.41% accuracy and a Matthews correlation coefficient (MCC) of 0.3717 on an independent dataset. This further demonstrates the value of pattern recognition in aptamer screening, by enabling meaningful predictions and offering insights to guide the design of target-specific aptamers.

#### 2.3.2. Deep Learning

In addition to traditional ML techniques, DL has emerged as a powerful extension due to its ability to automatically learn complex, non-linear representations from raw sequence data.

Convolutional neural networks (CNNs) are among the most frequently used DL architectures in aptamer discovery due to their ability to automatically extract meaningful sequence patterns. For example, DeepSELEX utilized a multi-class CNN trained on high-throughput SELEX (HT-SELEX) datasets, learning from changes in sequence enrichment over selection rounds to predict binding affinity. It modeled transcription factor binding across all SELEX cycles, outperforming methods that relied solely on final round data [60]. Another study employed a dual-branch CNN framework that independently processed aptamer sequences and target molecule fingerprints, enabling accurate prediction of binding affinities for small molecule targets such as ammonium. As validated by downstream docking, molecular dynamics (MD), and electrochemical sensing tests, this Smart-SELEX pipeline approach has effectively reduced the candidate pool [61].

Beyond CNNs, transformer-based architectures have also been applied to model aptamer-protein interactions. One such framework, AptaTrans, used pretrained sequence encoders and attention mechanisms to generate interaction maps between aptamers and protein targets [62]. The model demonstrated high predictive performance and was integrated into a generative pipeline that proposed novel aptamer candidates, several of which showed superior binding scores compared to SELEX-derived sequences in docking simulations.

In addition to discriminative models, generative DL approaches have also been explored to design aptamer sequences by modeling enrichment dynamics or structural features. For instance, a framework using Restricted Boltzmann Machines (RBMs) was trained on thrombin-binding sequences from SELEX to predict sequence enrichment and fitness, enabling the design of novel binders that were experimentally validated [63]. Another example is RaptGen, a variational autoencoder (VAE) framework using a profile hidden Markov model decoder to embed motif-specific representations from SELEX data [64]. It enables de novo generation, truncation, and affinity optimization of aptamer sequences through activity-guided latent space exploration. More recently, AptaDiff extended this concept by introducing a discrete diffusion model conditioned on motif-dependent latent embeddings learned via a VAE [65]. In the latent space, AptaDiff successfully generated aptamers with superior binding affinity, which, as validated by surface plasmon resonance (SPR) experiments, exceeded top SELEX-derived candidates in both binding kinetics and response unit signals.

Recurrent neural networks (RNNs) and their variants have also been explored in aptamer discovery, particularly for tasks involving sequential pattern learning and affinity prediction. These models are designed to process input as ordered sequences, capturing contextual dependencies across nucleotide chains. Long short-term memory (LSTM) networks and bidirectional LSTM (BiLSTM) architectures are among the most widely used variants that are especially effective at learning long-range interactions and sequence motifs relevant to aptamer folding and binding. In a related approach, a general regression neural network (GRNN), which is a memory-capable neural model conceptually similar to RNNs, was trained on amino acid-translated descriptors derived from aptamer sequences to predict binding affinities against influenza virus [66]. The model achieved strong predictive performance on both training and test sets, demonstrating the potential of sequence-aware neural models to uncover non-linear relationships between aptamer composition and binding strength, even in limited-data settings.

To further enhance performance, hybrid DL models are increasingly employed to combine the strengths of multiple architectures for aptamer discovery. For example, convolutional layers can capture local sequence motifs, while recurrent layers such as BiLSTM can model long-range dependencies across nucleotide chains. By integrating CNN, BiLSTM, and VAE modules, DeepAptamer is one such hybrid framework to jointly learn sequence patterns and structural properties such as DNA shape and loop regions [67]. Trained on early-round SELEX data, the model predicted aptamer enrichment with high accuracy and demonstrated strong correlation with experimental fitness. It also enabled the design of novel high-affinity aptamer candidates, several of which were validated through binding assays, thus offering a powerful alternative to labor-intensive enrichment cycles.

In this way, DL addresses the limitations of classical SELEX such as time, variability, and throughput. It also enables more targeted, high-resolution aptamer design that would be impractical to achieve through experimental screening alone. Table 1 below summarizes the discussed AI-driven aptamer discovery strategies. Taken together, these AI-driven approaches go beyond merely accelerating aptamer discovery. They expand the theoretical design space, uncover new sequence-structure-function relationships, and enable targeted optimization. As more curated datasets become available and model interpretability improves, these technologies are well-positioned to become integral components of next-generation aptamer engineering workflows.

**Table 1:** Summary of AI-Driven Aptamer Discovery Strategies.

AI Model Type	Specific Method/Tool	Input Features	Objective/Application	Validation/Outcome	References
Classical ML	SVM + structural descriptors	Molecular descriptors (e.g., topology, sequence motifs)	Classify aptamer binding affinity (e.g., streptavidin, cancer cells)	Nanomolar binders validated	[56,57]
	CountVectorizer + SVM	6-mer frequency vectors	Discriminate functional aptamers from background	AUROC = 0.998	[58]
	Random Forest + mRMR	Aptamer + protein sequence descriptors	Predict aptamer–target interactions	Accuracy: 77.41%, MCC: 0.3717	[59]
Deep Learning	DeepSELEX (CNN-based)	SELEX sequences across rounds	Predict enrichment cycle, binding potential	Outperformed DeepBind, BEESEM	[60]
	Smart-SELEX (CNN-based)	Aptamer + small molecule fingerprints	Predict small molecule binding (e.g., ammonium)	Validated by docking, MD, sensing	[61]
	AptaTrans (Transformer)	Aptamer–protein sequences	Predict/generate aptamers	Docking scores superior to SELEX aptamers	[62]
Generative DL	RBM (Restricted Boltzmann Machine)	SELEX-enriched sequences	Score enrichment and design aptamers	Experimental validation	[63]
	RaptGen (VAE-based)	Motif embeddings from SELEX	De novo generation, truncation, optimization	Latent space-guided design	[64]
	AptaDiff (Diffusion model)	VAE + motif-conditioned embeddings	Generate optimized aptamers	SPR: improved binding vs. SELEX	[65]
Sequence-aware DL	GRNN (RNN-like)	Amino acid-translated descriptors	Predict affinity (influenza aptamers)	R^2^ = 0.987 (train), 0.951 (test)	[66]
Hybrid DL	DeepAptamer (CNN + BiLSTM + VAE)	Sequence, DNA shape, loops	Predict enrichment, design aptamers	Validated high-affinity candidates	[67]

### 2.4. Structure-Based Modeling and Simulation

**Figure 2:**
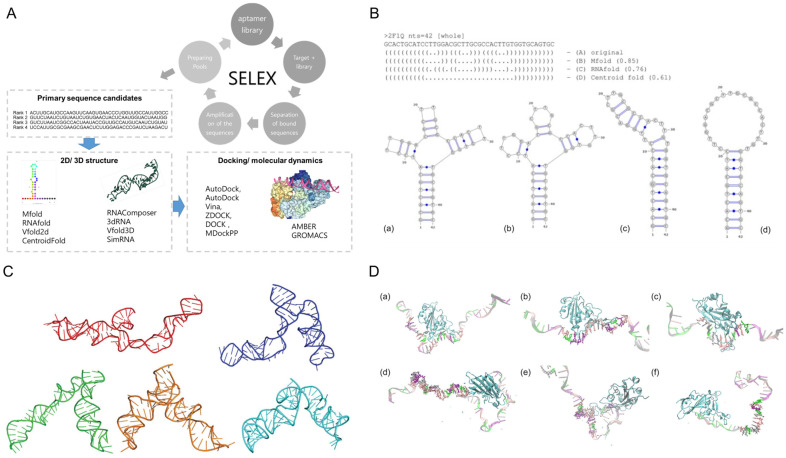
Structural-based modeling for rational aptamer selection. (**A**) Schematic overview of an in silico aptamer design pipeline, which typically integrates primary sequence selection, secondary and tertiary structure prediction, molecular docking, and molecular dynamics simulations to iteratively optimize binding affinity and structural stability. Figure reproduced from [41], licensed under CC BY 4.0. (**B**) Comparison of secondary structure predictions for the 2F1Q DNA aptamer generated by (**b**) Mfold, (**c**) RNAfold, and (**d**) CentroidFold, alongside (**a**) the experimentally resolved structure. The figure illustrates how different tools produce varying structural outputs from the same sequence, highlighting the importance of tool selection in aptamer fold prediction. Figure reproduced from [72], licensed under CC BY-NC-SA 4.0. (**C**) Representative RNA 3D structures predicted by 3dRNA/DNA, showing five clustered conformations derived from 500 sampled models [73]. Each structure represents the lowest-energy conformation from one cluster, illustrating structural variability in predicted aptamer folds under a template-based modeling approach. Copyright © 2023, Wiley Periodicals LLC. All rights reserved. (**D**) Initial docking poses of (**a**) Apt1, (**b**) Apt2, and (**c**) Apt3 with the SARS-CoV-2 spike RBD, generated using HDOCK. Each aptamer binds a distinct surface region of the RBD, providing a basis for subsequent MD simulations to evaluate binding stability and interaction profiles of (**d**) Apt1-RBD, (**e**) Apt2-RBD, and (**f**) Apt3-RBD. Figure reproduced from [79], licensed under CC BY 4.0.

In addition to data-driven strategies, structure-based computational methods have become essential tools in aptamer research. Figure 2A represents the typical workflow of in silico structure-based aptamer selection. By simulating aptamer-target interactions in three dimensions, these approaches offer atomistic insight into binding conformations, energetics, and structural dynamics. Such information supports rational aptamer design and optimization, particularly in applications requiring high stability, specificity, or device integration.

#### 2.4.1. Secondary and Tertiary Structure Prediction

The function of aptamers is intrinsically linked to their ability to fold into specific secondary and tertiary structures that form the binding pockets required for high-affinity and high-specificity interactions. Subtle changes in loop regions, stem length, or overall conformation can markedly affect binding efficiency, making accurate structural characterization a prerequisite for rational design and application [68,69].

Secondary structure prediction tools, such as Mfold, estimate thermodynamically favorable base-pairing interactions; therein providing insights into stem-loop motifs, bulges, and other structural features essential for aptamer function [70]. These tools are widely used for designing or refining aptamer candidates prior to higher-order structural modeling. In one study, secondary structures generated using Mfold were encoded and analyzed to identify conserved features among DNA aptamers derived from CompELS screening [71]. To demonstrate how structure-based metrics can complement sequence-based selection and to reveal conserved motifs that guide prioritization of unique candidates, the authors used secondary structure strings to classify aptamers into families based on folding similarity, and applied position-specific scoring and multiple secondary structure string alignment (MSS$A) methods to examine structural conservation across candidates.

To evaluate tool performance, a systematic benchmarking study assessed Mfold, RNAfold, and CentroidFold on DNA aptamers with experimentally validated crystal structures (Figure 2B). The study found that most DNA aptamer folds could be reasonably predicted, although complex features such as G-quadruplexes and pseudoknots often reduced prediction accuracy. Notably, RNAfold showed improved performance on G-quadruplex-containing structures when G-quadruplex modeling was enabled. However, performance was sequence-dependent, and G-quadruplex reconstruction often remained challenging even with these advanced tools [72].

Tertiary structure modeling builds on secondary structure predictions to provide three-dimensional (3D) insights into aptamer folding. As illustrated in Figure 2C, 3dRNA/DNA is one of the available platforms that enables integrated tertiary modeling of both RNA and DNA aptamers through template-based reconstruction and fragment assembly [73]. It supports loop generation, structure refinement, and scoring via 3dRNAscore, enabling efficient prediction and evaluation of aptamer conformations for downstream applications. RNAComposer is another fully automated web server for RNA 3D structure prediction based on user-specified secondary structures. It uses a fragment assembly approach supported by an extensive 3D motif dictionary and can incorporate experimental distance restraints for higher accuracy. Benchmarking against known structures and use in RNA nanotechnology applications have demonstrated its reliability and versatility in modeling complex RNA architectures [74]. A structure prediction pipeline integrating Mfold, Assemble2, and MD simulations has been used to reconstruct 3D conformations of hairpin-forming ssDNA aptamers, yielding conformations consistent with experimentally determined structures [75]. This modeling approach provides a practical route for visualizing aptamer folding and supports downstream applications such as biosensor interface design.

#### 2.4.2. Docking and Binding Simulations

Once aptamer structures are generated, molecular docking and MD simulations serve as essential tools for evaluating aptamer-target interactions at atomic resolution. Docking predicts favorable binding orientations based on energetics, while MD captures the temporal evolution of these interactions, providing insight into conformational dynamics, stability, and binding mechanisms.

Docking tools such as AutoDock, HDOCK, and ZDOCK are commonly employed to simulate aptamer binding to proteins or small molecules by predicting favorable binding orientations and calculating scoring functions based on energy minimization. The effectiveness of molecular docking using AutoDock Vina was evaluated on eight aptamers targeting testosterone and its analogs, under varying ionic and temperature conditions, with the aim of assessing binding affinity and structural complementarity. The study identified TESS1 as the most stable and affine candidate based on the number of interactions (e.g., hydrogen bonds, pi-alkyl) and docking ΔG values, which were further validated through relative capture experiments [76]. AutoDock Vina 1.2.0 introduced several enhancements over its earlier versions, including support for the AutoDock4.2 scoring function, hydrated docking, and macrocycle sampling, all integrated within a faster Monte Carlo-based search algorithm. These additions significantly improved docking accuracy for complex systems such as zinc-metalloproteins and macrocyclic ligands, while also enabling batch docking and scripting through Python bindings for high-throughput aptamer screening [77]. These advancements in docking technology have paved the way for more efficient screening pipelines in aptamer research.

In a separate study, a generative model called Apta-MCTS, which combined Monte Carlo Tree Search with aptamer-protein interaction classifiers, was proposed to generate RNA aptamers for protein targets. Candidate sequences generated in silico were evaluated using ZDOCK docking simulations, and results showed that the predicted aptamers achieved docking scores comparable to or exceeding those of experimentally derived or previously predicted aptamers across multiple targets [78].

Integrated docking-MD approaches have also been employed besides standalone docking studies. For instance, docking using HDOCK combined with 1000 ns MD simulations was conducted to evaluate the binding of three DNA aptamers to the SARS-CoV-2 spike protein RBD. As supported by root-mean-square distance (RMSD), hydrogen bond occupancy, and molecular mechanics Poisson–Boltzmann surface area (MM/PBSA) energy decomposition analysis, Apt1 showed the strongest and most consistent binding. These findings, as shown in Figure 2D, highlight the potential of docking-MD pipelines in identifying structurally stable aptamer-target complexes for diagnostic applications [79]. Another relevant study on DNA aptamers targeting the SARS-CoV-2 nucleocapsid protein were identified and validated using both docking and MD simulations to uncover critical binding interactions [80]. The tNSP3 aptamer was docked to predicted epitope regions of the N protein (AA10 and AA21 peptides), and the resulting complexes were refined using 200 ns MD simulations. The binding was primarily mediated through nucleotides located in the aptamer loop, and simulations revealed stable complexes with consistent hydrogen bonding networks and low dissociation behavior, highlighting the structural basis for its high-affinity binding. This integrated approach informed the rational design of diagnostic aptamers with nanomolar sensitivity in biosensing applications.

While docking provides a static snapshot of binding configurations, MD simulations offer dynamic validation by modeling structural fluctuations and interaction stability over time. Platforms such as GROMACS and Assisted Model Building with Energy Refinement (AMBER) allow researchers to assess binding-induced conformational shifts, aptamer flexibility, and solvent interactions. MD simulations, for example, were performed using GROMACS to refine 3D RNA aptamer structures initially predicted by Mfold and RNAComposer. The study demonstrated that MD simulations could improve aptamer conformations by stabilizing non-canonical loop structures and aligning predicted geometries more closely with NMR-resolved reference structures. RMSD analyses confirmed that representative MD-derived models better reflected native structures, especially when clustering was used to extract stable conformations [81].

In another example of MD simulations, cooperative binding mechanisms and key structural transitions over microsecond timescales were investigated [82]. In particular, AMBER force fields (OL15 for DNA and GAFF for AMP) were applied within GROMACS to simulate single- and dual-site recognition in an AMP-binding DNA aptamer. Enhanced sampling techniques revealed site-specific differences in binding stability and electrostatic interactions.

A coarse-grained (CG) molecular simulation approach combining the STUN-BH-DMD method and the MARTINI force field was used to predict the most stable complex between a DNA aptamer (EpA) and its target EpCAM. The study revealed that binding within a pocket-like structure on EpCAM was energetically more favorable, with MD simulations confirming aptamer flexibility and stability under aqueous conditions. This workflow highlights how advanced dynamic modeling techniques can guide aptamer optimization through structural and energetic insights [83].

Collectively, structure-based simulation techniques demonstrate complementarity to sequence- and structure-prediction tools by validating aptamer-target interactions in silico, guiding sequence refinement, and supporting the rational design of high-performance aptamers for diagnostic and therapeutic use.

#### 2.4.3. Binding Visualization

A variety of software tools are available to display aptamer-target docking complexes and analyze the results of MD simulations.

Protein–Ligand Interaction Profiler (PLIP) is a widely used visualization and analysis platform for identifying non-covalent interactions within biomolecular complexes. It is now expanded to support DNA and RNA targets, automatically detecting hydrogen bonds, π-stacking, salt bridges, and other contacts. PLIP outputs interactive 3D visualizations, including PyMOL sessions and JSmol renderings, which are especially useful for aptamer-ligand or aptamer-protein interaction analysis following docking or MD simulations [84].

LigPlot+ is commonly used to generate 2D interaction maps that summarize the key residues involved in binding, while PyMOL provides high-resolution 3D visualization for spatial analysis. For example, one study performed docking simulations using the Molecular Operating Environment (MOE) software (MOE version 2016.0802) and binding site visualization was carried out using both PyMOL and LigPlot+ to evaluate aptamer interactions with botulinum neurotoxin type C (BoNT/C). Visualization clearly demonstrated that selected aptamer pairs bound to distinct, non-overlapping regions on the target protein, supporting their use in a sandwich assay format for sensitive detection [85].

Biovia Discovery Studio is another established platform for visualizing and analyzing molecular data. In one study, PLIP was used to extract non-covalent contacts from docking complexes involving programmed death-ligand 1 (PD-L1) and DNA aptamers. Discovery Studio was subsequently used to visualize the structures and generate labeled diagrams of binding interactions [86]. Similar research combined PyMOL and Discovery Studio to examine electrostatic interactions, hydrogen bonding, and binding orientation between aptamers and protein PPIA, thereby validating binding pose complementarity [87]. In one recent work, BIOVIA Discovery Studio was employed in a study for both docking preparation and interaction analysis between DNA aptamers and the prostate-specific antigen (PSA) [88].

Together, these results indicate that visualization tools enhance structural interpretation and streamline downstream analysis in aptamer research. By integrating structure prediction, docking, molecular simulation, and visualization, structure-based modeling workflows enable researchers to rationally design aptamers with improved binding performance, offering a strong complement to both experimental selection and AI-driven discovery strategies.

## 3. Aptasensors: Design, Functionalization and Computational Enhancements

### 3.1. Biosensor Architecture: Electrochemical and Beyond

Biosensors, analytical devices that integrate a biological recognition element with a physicochemical transducer, have undergone significant advancements in recent years, driven by the demand for higher sensitivity, specificity, and real-time diagnostic capabilities. The architecture of a biosensor refers to the structural and functional design that dictates how the biological recognition element interacts with the transducer to produce a measurable signal. This design is crucial as it determines the sensitivity, specificity, response time, and overall performance of the biosensor.

Among the various classes of biosensors, electrochemical, optical, acoustic, and thermal biosensors stand out due to their versatility and wide-ranging applications. The continuous evolution of biosensor architecture is characterized by the integration of nanomaterials [89,90], enhanced surface functionalization utilizing techniques like ellipsometry and atomic force microscopy [91], and the application of advanced signal transduction techniques, with a notable example being CRISPR-based bioanalytics [92]. The employment of such strategies is leading to cutting-edge aptasensor platforms with unprecedented performance.

#### 3.1.1. Electrochemical Aptasensors

Electrochemical aptasensors (E-AB) that employ aptamers as selective biorecognition elements have increasingly widespread use in biosensing due to their high sensitivity, fast response times, and low operational cost. These systems detect target binding events by monitoring changes in electrical signals generated upon aptamer-analyte interactions. [93]. Recent innovations have prioritized the refinement of electrode materials through the incorporation of nanostructured carbons like carbon nanotubes and graphene, metal nanoparticles such as gold and platinum, and metal–organic frameworks (MOFs), all of which markedly improve electron transfer kinetics and expand the surface area available for aptamer immobilization [94,95,96,97]. These enhancements have enabled the development of flexible, wearable E-AB capable of monitoring and detecting stress-related biomarkers like cortisol and anticancer medications in real-time [98,99]. Further improvements in the stability and specificity of aptamer immobilization stem from advanced surface engineering, including self-assembled monolayers (SAMs) and conductive polymer coatings that strengthen performance in complex sample conditions [100,101].

#### 3.1.2. Optical Aptasensors: Innovations in Light-Mediated Detection

Optical aptasensors take advantage of the interaction between light and matter to achieve highly sensitive, label-free detection of target analytes. A diverse array of spectroscopic detection methods that includes fluorescence resonance energy transfer (FRET), SPR, localized surface plasmon resonance (LSPR), and colorimetric assays has been extensively utilized to advance analytical capabilities like specific target recognition in this field [102,103,104]. Current advancements in plasmonic nanostructures, such as gold and silver nanoparticles, have enhanced the signal amplification of SPR and LSPR platforms, which enables high precision detection of low-abundance biomarkers [105]. Likewise, linking FRET with DNA aptasensors has allowed for even greater sensitivity in biomarker detection. This is exemplified by the work of Ghosh et al. (2020) who demonstrated that constructing a FRET-based DNA aptasensor enabled the intracellular detection of Tumor necrosis factor alpha (TNF-α) [106]. Fluorescent aptasensors have similarly advanced through the development of QDs and upconversion nanoparticles, which offer superior photostability and high signal-to-noise (S/N) ratios [107,108]. Hybrid optical systems, integrating multiple detection methods, have also been developed to improve versatility, enabling analyte detection of small biomolecules ranging from adenosine triphosphate (ATP), and TNF-α to more complex diagnostic applications in cancer [106,109,110].

#### 3.1.3. Acoustic Aptasensors: Real-Time, Label-Free Monitoring

Acoustic aptasensors, including quartz crystal microbalance (QCM) and surface acoustic wave (SAW) devices, exploit changes in acoustic wave properties such as frequency, amplitude, or phase, which results from the binding of the target analyte to the aptamer-functionalized surface [111,112]. Such platforms are inherently label-free and provide real-time monitoring of molecular interactions, making them particularly valuable for studying binding kinetics and affinity [113]. Breakthroughs using micro- and nano-scale surface modifications, such as nanostructured gold coatings and hybrid organic-inorganic interfaces, which enhance acoustic wave propagation and signal transduction [114,115,116,117], have led to enhanced selectivity and sensitivity.

Furthermore, the integration of microfluidic systems with SELEX for aptamer selection has led to improved detection performance and significant advances in lab-on-a-chip technology [118,119,120]. Thus, the blending of acoustics and microfluidics with the aptamer selection processes indicates that merging of detection techniques offers a novel means of advancing biosensor architecture. This is evidenced by the work of Chen et al. (2020) who combined Ag10NPs nano-biosensors with a PDMS/glass microfluidic biochip to develop a rapid (~1 h) detection method for antibiotic resistant *Klebsiella pneumoniae* carbapenemase 2 (KPC-2)-expressing bacteria [121].

#### 3.1.4. Thermal Aptasensors: Harnessing Calorimetric Detection

Thermal aptasensors represent a burgeoning frontier in biosensor technology, leveraging the detection of temperature changes resulting from aptamer-target interactions. These sensors offer a label-free approach to monitoring biochemical events, capitalizing on the thermodynamic shifts that occur upon binding [122].

Ongoing developments have significantly enhanced the sensitivity and versatility of thermal aptasensors. One notable innovation is the development of microcalorimetric sensors with enhanced thermal sensitivity, capable of detecting subtle exothermic or endothermic reactions with high precision [123]. These microcalorimetric systems utilize heat flux sensors integrated into a microfluidic chip, allowing for rapid and accurate measurement of heat changes in confined reaction volumes. Such an approach minimizes heat loss and enhances detection sensitivity, making it highly applicable to thermal aptasensors.

Moreover, the development of melting aptasensors has emerged as a promising approach to thermal detection. These devices monitor shifts in the thermal stability (melting temperature, T_m_) of aptamers upon target binding, providing a dual-mode detection strategy. For instance, thermal fluorescence analysis (TFA) utilizes dual-labeled aptamers that exhibit distinct fluorescence signals depending on their conformational state. Such systems have demonstrated high sensitivity and reusability, allowing for thousands of signaling and regenerating cycles without significant loss of performance [124]. The integration of advanced thermal insulation materials, precise temperature control systems, and optimized microfluidic designs has further improved the performance of thermal aptasensors, making them a versatile tool for detecting a wide range of analytes. Additionally, the development of dual-mode colorimetric and photothermal aptasensors has further expanded the capabilities of thermal aptasensors. In a recent study, Lee et al. (2024) [125] developed a dual-mode aptasensor using chitosan-stabilized platinum nanoparticles (CS/PtNPs) for the detection of kanamycin. The combination of colorimetric and photothermal modes achieved a detection limit of 0.04 μM and 0.41 μM for kanamycin, respectively [125]. Such dual-mode systems not only enhance sensitivity and specificity, but also enable flexible detection strategies, making them highly versatile for diagnostic applications.

The integration of advanced materials has further enhanced the sensitivity and specificity of thermal aptasensors. One pertinent example is how the incorporation of nanomaterials with high thermal conductivity and specific heat capacity has improved the detection of minute thermal changes associated with target binding events [126]. These innovations in thermal aptasensor technology reflect a broader trend toward enhancing biosensor performance through the integration of innovative detection strategies, material enhancements, and microfluidic designs. The continued evolution of thermal aptasensors underscores their potential as versatile tools in biosensing applications, such as in clinical diagnostics.

Evidently, the architecture of a biosensor fundamentally shapes aptasensor performance by influencing sensitivity, specificity, and stability. Platform choice—electrochemical, optical, acoustic, or thermal—determines surface properties and signal transduction, directly affecting aptamer immobilization and activity. Thus, effective immobilization is essential to maintain aptamer structure and ensure accurate target recognition, enabling reliable and high-performance sensing. A comparative summary of key aptasensor platforms, including their operational methods, constituent materials, primary applications, and performance metrics, is provided in Table 2, offering a consolidated overview of how sensor architecture shapes diagnostic utility.

**Table 2:** Comparative Overview of Aptasensor Architectures.

Aptasensor Type	Key Method	Materials	Application	Performance Metrics	References
Electrochemical (E-AB)	Electrode modification; nanostructured interfaces	CNTs, graphene, Au/Pt nanoparticles, MOFs; SAMs; conductive polymers	Enhance electron transfer; real-time biomarker monitoring	Fast response; wearable and complex sample compatibility; high sensitivity via nanomaterials	[93,94,95,96,97,98,99,100,101]
Optical	Light–matter interactions (FRET, SPR, LSPR, colorimetry)	Plasmonic nanostructures (Au, Ag NPs); QDs; upconversion NPs	Ultrasensitive, label-free biomarker detection; multiplex cancer diagnostics	High sensitivity; improved photostability and S/N; versatile for low-abundance biomarkers	[102,103,104,105,106,107,108,109,110]
Acoustic	QCM and SAW with nano/micro surface modification; microfluidics integration	Nanostructured Au coatings; hybrid organic–inorganic surfaces; PDMS/glass chips; Ag10NP biosensors	Label-free, real-time binding analysis; lab-on-a-chip diagnostics	Real-time monitoring; Enhanced acoustic wave propagation and signal transduction	[111,112,113,114,115,116,117,118,119,120,121]
Thermal	Calorimetric detection; melting aptasensors; dual-mode sensing	Microcalorimetric sensors; dual-labeled aptamers; CS/PtNPs; advanced thermal insulation	Detect thermodynamic binding; dual-mode colorimetric and photothermal detection	High sensitivity; reusability (TFA cycles); versatile for clinical applications	[122,123,124,125,126]

### 3.2. Key Steps in Aptamer Immobilization

Aptamer immobilization is a critical process in the development of aptasensors, directly influencing their sensitivity, specificity, and stability. Immobilization ensures that aptamers are securely attached to the sensor surface while maintaining their ability to recognize and bind to target molecules. Achieving an optimal immobilization strategy requires a careful balance between strong, stable attachment and the preservation of aptamer conformational flexibility, which is essential for effective target binding. Some key elements requisite to enhancing sensor performance are surface chemistry, molecular orientation, and spatial distribution of immobilized aptamers. Aside from covalent bonding, the preferred method of aptamer immobilization due to its superior stability and reproducibility, other promising strategies include physical adsorption, electrostatic interactions, affinity-based immobilization, and computational optimization approaches. Each of these methods contributes uniquely to improving aptamer orientation, functional accessibility, and overall sensor performance depending on the application context.

#### 3.2.1. Covalent Immobilization Strategies

Covalent immobilization stands out as one of the most reliable methods for attaching aptamers to sensor surfaces due to its stability and resistance to environmental fluctuations. This method involves the formation of strong chemical bonds between functional groups on the aptamer and complementary reactive groups on the sensor surface [127]. Among the most used chemistries are thiol-gold interactions, where the strong affinity between thiol (-SH) groups and gold surfaces is exploited to form a stable SAM [128,129]. This approach is extensively used in electrochemical and SPR aptasensors, where gold electrodes provide a conductive and biocompatible platform. Optimizing the density of thiol-modified aptamers has been shown to significantly enhance target binding by minimizing steric hindrance [130,131]. Indeed, Simon et al. (2020) illustrated the importance of aptamer surface density and showed there is a strong correlation between target binding efficiency and the size of the target analytes [131].

**Figure 3:**
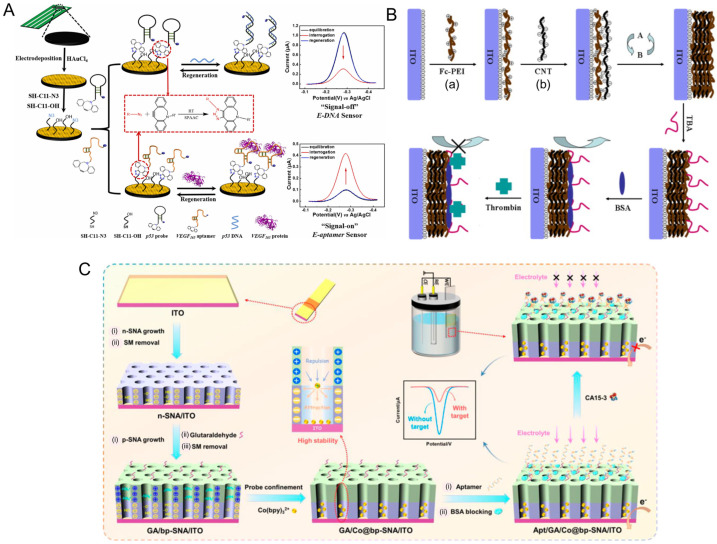
Advances in aptamer immobilization strategies. (**A**) Strain-Promoted Azide-Alkyne Cycloaddition (SPAAC) mediated surface grafting for VEGF_165_ aptamer or p53 complementary probe immobilization and electrochemical biosensing [133]. Copyright © 2021, Elsevier B.V. All rights reserved. (**B**) Layer-by-Layer (LbL) surface assembly using ferrocene-appended poly(ethyleneimine) (Fc-PEI) and carbon nanotubes (CNTs) for aptamer immobilization and thrombin biosensing [134] Copyright © 2010, Elsevier B.V. All rights reserved. (a) Primary electrode surface coating of Fc-PEI. (b) Secondary coating of CNTs. (**C**) Bipolar-silica nanochannel array film (bp-SNA) composed of a negatively charged inner layer (n-SNA) and a positively charged outer layer where amino-modified CA15-3 aptamers were immobilized after aldehyde derivatization on the outer surface. Figure reproduced from [135], licensed under CC BY 4.0.

Another widely used approach is carbodiimide chemistry, which employs 1-ethyl-3-(3-dimethylaminopropyl) carbodiimide (EDC) and N-hydroxysuccinimide (NHS) to covalently link carboxyl-modified aptamers to amine-functionalized surfaces [127,132]. This method is versatile, enabling aptamer immobilization on a wide range of substrates, including carbon, graphene, and silica [132]. More advanced methods, such as click chemistry, provide highly specific and efficient covalent attachment through azide-alkyne cycloaddition. As a proof-of-principle, Fan et al. (2022) demonstrated that utilizing copper-free Strain-Promoted Azide-Alkyne Cycloaddition (SPAAC) offered a less-expensive biosensor fabrication method with good stability, reproducibility, and a low limit of detection when tested against p53 DNA and vascular endothelial growth factor (VEGF) 165 protein (Figure 3A) [133]. In turn, this technique offers a complimentary method of optimizing probe density and limiting steric hindrance, indicating that it is valuable as an immobilization strategy that can be utilized to overcome issues with aptamer surface density.

#### 3.2.2. Physical and Electrostatic Immobilization

Physical immobilization relies on non-covalent interactions such as electrostatic forces, van der Waals interactions, and hydrophobic effects to achieve aptamer immobilization. This approach is simple and cost-effective, making it suitable for disposable biosensors where rapid and reversible attachment is desirable. Carbon-based platforms, including graphene, carbon nanotubes, and carbon black, are commonly used as substrates due to their high surface area and excellent conductivity [136]. Enhancing these carbon substrates with metal nanoparticles or conductive polymers has been shown to significantly improve signal transduction and stability [137]. An advanced form of physical adsorption, known as layer-by-layer (LbL) assembly shown in Figure 3B, enables the sequential deposition of oppositely charged materials, providing precise control over aptamer density and surface coverage with a unique, stable membrane property [134]. This approach has been used to fabricate multilayered aptasensors with enhanced sensitivity and robustness. In a recent study, Patil et al. (2024) designed an Ag-incorporated cobalt-succinate metal–organic framework for the immobilization of thiol- and amino-modified DNA aptamers with an impressive limit of detection of 0.3 nM, illustrating how LbL assembly can be applied to improve biosensing capacity [138].

Electrostatic immobilization, a subtype of physical immobilization, offers a versatile and efficient approach for attaching nucleic acid aptamers to sensor surfaces, leveraging the inherent negative charge of the phosphate backbone of aptamers and the attraction to positively charged substrates. This method provides several advantages, including simplicity, cost-effectiveness, and the ability to achieve high surface density without requiring complex chemical modifications. Notably, polyaniline and chitosan-modified electrodes have emerged as popular substrates for electrostatic immobilization due to their positive charge and biocompatibility. In an electrochemiluminescence (ECL) aptasensor for Hg^2+^ detection, a composite of poly(aniline-luminol)/graphene oxide/chitosan (P(ANi-Lu)/GO/CS) demonstrated effective electrostatic adsorption of negatively charged aptamers, underscoring the utility of chitosan as a positively charged immobilization matrix [139].

The effectiveness of electrostatic immobilization can be further enhanced by optimizing the ionic strength and pH of the immobilization solution. These factors influence the density and orientation of immobilized aptamers, directly impacting their ability to recognize target molecules. Research has shown that the binding affinity of aptamers is highly sensitive to changes in ionic strength and pH, with optimal conditions significantly improving target accessibility [140].

Beyond simple charge-based interactions, electrostatic immobilization on nanostructured substrates has shown remarkable promise. MOFs and conductive polymers provide high surface area and favorable electrostatic environments, facilitating the immobilization of aptamers and improving signal transduction. Novel approaches include a bipolar silica nanochannel array (bp-SNA) with dual charged layers (n-SNA and p-SNA) that was used to construct an advanced aptasensor, achieving stable and high-density aptamer immobilization due to the complementary electrostatic interactions of the dual layers (Figure 3C). This configuration enabled reagentless electrochemical detection of carbohydrate antigen 15-3 (CA15-3) with excellent sensitivity and stability [135].

These studies illustrate that electrostatic immobilization is a highly adaptable method capable of achieving high aptamer density, maintaining aptamer functionality, and enhancing sensor performance. Its versatility ranges from simple charge-based adsorption on conductive polymers to sophisticated dual-layer systems that leverage complementary electrostatic interactions. Such approaches not only enhance sensitivity, but also provide a foundation for the development of highly optimized aptasensor platforms that combine stability, reusability, and high analytical performance.

#### 3.2.3. Affinity-Based Immobilization Techniques

Affinity-based immobilization offers a sophisticated and highly specific approach to attaching aptamers to sensor surfaces, exploiting the principles of biological recognition to achieve stable and oriented attachment. Among these techniques, the biotin-streptavidin interaction remains the most widely used due to its exceptionally high binding affinity. Importantly, this strong non-covalent interaction is inherently irreversible, and thus limits the potential for sensor regeneration. To address this, innovative methods have been developed to achieve reversible biotin-streptavidin interactions, such as the use of Ni-nitrilotriacetic acid (Ni-NTA) with 6xHis-tagged streptavidin, enabling the regeneration of sensor surfaces without compromising stability [141]. Moreover, a predictive model for controlling streptavidin coverage on biotinylated surfaces has been established, allowing for precise regulation of surface density and enhancement of biosensor performance [142].

Antibody–aptamer hybrid systems present another versatile affinity-based approach, combining the high specificity of antibodies with the stability and synthetic flexibility of aptamers [143]. Application of these hybrid systems can be found in dual-mode biosensors, such as those for salivary cortisol detection, where the antibody-aptamer sandwich model enables simultaneous target recognition and signal amplification [144]. This dual-mode approach significantly improves sensitivity and specificity, making it highly valuable for diagnostic applications.

DNA hybridization-based immobilization offers precise control over aptamer orientation and density by exploiting the complementary nature of nucleic acid strands [145]. This method can be further refined using partially complementary strands, which promote the formation of secondary structures such as G-quadruplexes to enhance target binding affinity [146]. Such controlled assembly of aptamers improves target recognition and enhances the overall performance of the biosensor.

Peptide-based immobilization techniques represent another innovative affinity-based strategy. By utilizing peptide sequences with specific binding domains, aptamers can be immobilized in a modular and adaptable manner. This approach has been applied to design versatile biosensor platforms capable of detecting a wide range of targets with high sensitivity [147]. Evidently, affinity-based immobilization methods offer exceptional flexibility and specificity, making them invaluable in the development of advanced aptasensor technologies. By facilitating modular design, enhanced regeneration capabilities, and precise molecular orientation, these techniques are likely to play an increasingly central role in advancing biosensor reliability and customizability across diverse diagnostic applications.

#### 3.2.4. Computational Optimization of Immobilization Strategies

Computational optimization has emerged as a transformative approach for enhancing aptamer immobilization strategies, providing insights into the most effective surface chemistries, optimal aptamer density, and consistent target accessibility. MD simulations have been extensively used to study the conformational flexibility of surface-bound aptamers, revealing how immobilization orientation affects target binding efficiency [148]. Simulations of this sort provide a detailed understanding of the structural dynamics of aptamers, enhancing the identification of optimal attachment strategies that maintain target recognition capability.

Computational docking studies further enhance this understanding by predicting the binding interactions between aptamers and their targets, offering a rational basis for selecting appropriate immobilization methods [149]. For example, in a 2021 investigation by Jeddi et al., an all-atom MD simulation was used to optimize the attachment configuration of an anti-MUC1 aptamer on a silica biosensor, identifying conditions that maximized target binding and minimized non-functional aptamer collapse [150]. This approach enabled the rational design of a highly efficient aptamer-based biosensor with improved performance through stabilization of an upright configuration.

Utilizing ML has allowed researchers to illuminate novel data-driven solutions that have led to marked improvements in aptamer optimization. As evinced through the use of diffusion models, ML offers a viable method for generating and optimizing aptamers with superior binding affinity [65]. Along similar lines, leveraging deep neural networks and pre-trained encoders has enabled better prediction of aptamer–protein interactions by incorporating insights from tertiary structure analysis. Expanding this toolkit, non-SELEX methods such as AptaBLE [151] harness large language models to identify high-affinity binding partners and design novel aptamer sequences, showcasing improved efficacy in de novo aptamer development. AptaBLE further illustrates the potential of ML in aptamer optimization, including applications in SELEX optimization and prediction of aptamer-protein interactions. Although these ML approaches primarily focus on aptamer sequence optimization and binding prediction, their capacity to generate high-affinity aptamers suggests they may also hold promise for optimizing immobilization strategies by enabling the rational selection of aptamers with improved surface-binding characteristics.

As highlighted in a review by Yu et al. (2023), the integration of computational tools with post-SELEX optimization methods enables the continuous improvement of aptamer performance, demonstrating another approach in advancing aptamer immobilization through computation [152]. Such computational approaches provide a robust framework for optimizing aptamers that allows researchers to move beyond empirical methods to achieve precise and reproducible sensor designs.

Aptamer immobilization critically shapes the sensitivity, specificity, and stability of aptasensors. Methods such as covalent bonding, physical adsorption, and affinity-based approaches offer varying benefits in stability, reversibility, and orientation control. Computational tools, ranging from molecular modeling to ML, are now streamlining design and optimization. Moving forward, integration of these strategies will be key to developing adaptable, high-performance diagnostic platforms.

### 3.3. Computational Optimization and Design of Biosensors

The integration of computational methodologies has revolutionized biosensor design, providing a robust framework for enhancing sensor performance, sensitivity, and specificity. These approaches, which span ML, MD simulations, hybrid computational models, and advanced predictive frameworks, enable the precise selection, modification, and optimization of aptamers for diverse sensing applications. This section explores recent advancements in computational strategies that have significantly improved biosensor performance, with a focus on aptamer design, target recognition, structural refinement, and signal enhancement. Emerging evidence indicates computational tools are being harnessed to streamline aptamer engineering and elevate biosensor functionality across diverse platforms.

#### 3.3.1. Machine Learning-Driven Aptamer Optimization

ML models have transformed aptamer selection and biosensor optimization by automating complex data analyses and identifying critical factors influencing sensor performance. Advanced algorithms such as SVM, RF, CNNs, and RNNs are widely employed for predictive modeling in biosensors [67,153,154,155]. A notable illustration is AptaNet, a DL approach combining CNNs and RNNs that has demonstrated superior performance in predicting aptamer-protein interactions by learning intricate sequence-structure relationships from high-throughput screening data [153].

Through Bayesian optimization, AptaDiff is capable of engineering aptamers without relying solely on high-throughput sequencing data, resulting in enhanced binding efficiency to target proteins [65]. In a complementary advance, AptaTrans’ capacity for structure-aware representation learning allowed for refined in silico screening of aptamer candidates beyond conventional alignment-based methods [55]. The value of utilizing DL approaches to advance aptamer design for diverse targets is further exemplified by AptaBLE’s large language model [151]. In a notable advancement, DeepAptamer, a hybrid model integrating CNNs and BiLSTM, bypasses the limitations of traditional SELEX by accurately predicting high-affinity aptamers from unenriched SELEX pools. This reduces the need for 20–30 iterative selection rounds and offers an effective means of identifying key binding motifs critical for target recognition [67]. In addition, Bashir et al. (2021) showcased the synergistic potential of combining ML with particle display (PD), leading to an 11-fold improvement in the identification of high-affinity aptamers compared to random perturbations [156]. This approach not only enhanced the discovery of novel, high-affinity aptamers, but also facilitated the design of truncated aptamers that were 70% shorter and exhibited superior binding affinity (i.e., 1.5 nM) compared to the best experimental candidate [156]. These developments underscore the transformative role that ML plays in streamlining aptamer discovery and optimizing binding performance. Ultimately, paving the way for more efficient and targeted biosensor development.

#### 3.3.2. Molecular Dynamics Simulations for Structural Insights

MD simulations offer critical insights into the structural dynamics of aptamers and their interactions with target molecules. These simulations model the conformational flexibility and binding kinetics of aptamers, revealing the impact of environmental factors such as ionic strength, temperature, and solvent conditions on target recognition [157]. Enhanced sampling techniques, such as umbrella sampling and metadynamics, further improve the accuracy of MD simulations, providing a detailed understanding of the transition pathways between aptamer states. Such insights are essential for optimizing aptamer structure and function, ensuring stable and high-affinity interactions with target molecules. Evidencing this, Ramasanoff et al. (2023) employed umbrella sampling to elucidate the selective binding mechanism of an adenosine-specific DNA aptamer, revealing that the disordered structure of the aptamer’s internal loop and its network of hydrogen bonds are critical for stabilizing the binding site, thus preventing barrier-free penetration of ligands [158].

Additionally, the application of metadynamics combined with alchemical free energy calculations has been shown to accurately predict the binding affinity of a theophylline-RNA aptamer complex, which was in direct alignment with experimental values [159]. Other applications of MD simulations aimed at investigating DNA aptamers targeting the spike protein of SARS-CoV-2, revealed that aptamer binding can effectively block the transition between the closed and open conformations of the spike protein, thereby impeding viral entry into host cells [160]. By providing accurate binding estimates and revealing the mechanistic underpinnings of conformational transitions, advanced sampling methods like MD significantly enhance our understanding of aptamer-target complex dynamics that are crucial for biosensor performance.

#### 3.3.3. Predictive Models for Binding Efficiency

The development of predictive models for aptamer binding efficiency is critical for optimizing sensor performance. These models utilize a combination of thermodynamic calculations, sequence-structure relationships, and ML to forecast the affinity and specificity of aptamers for their targets. Thermodynamic models, such as the Minimum Free Energy (MFE) model, are commonly used to predict the most stable secondary structure of aptamers, providing insights into optimization strategies and new aptamer design [40].

Advanced models like SPOT-RNA and MXfold2 employ DL to enhance secondary structure prediction, capturing complex motifs such as G-quadruplexes and pseudoknots. Notably, SPOT-RNA, comprising an ensemble of two-dimensional deep neural networks combined with transfer learning from a large bpRNA dataset, has been shown to significantly outperform traditional folding-based algorithms by accurately predicting base-pairing interactions, including noncanonical and non-nested (i.e., pseudoknot) base pairs [161]. This advancement enables precise modeling of RNA secondary structures, including complex motifs stabilized by tertiary interactions. Further improvements have been achieved using evolutionary profiles and mutational coupling. A case in point is the work by Singh et al. (2021), where the model’s predictive accuracy was further enhanced by incorporating artificial homologous sequences generated through deep mutational scanning. This method achieved an F1-score of >0.8 for 14 of 16 RNAs tested, capturing both secondary and tertiary interactions such as pseudoknots and lone base pairs, illustrating that it is a robust tool for RNA structural prediction [162].

In addition, a comparative analysis of secondary structure prediction tools revealed that models like MXfold2 and SPOT-RNA consistently outperform other approaches, particularly for single-stranded nucleic acids (ssNAs), where they demonstrate superior accuracy in predicting complex structures like pseudoknots [163]. Predictive modeling approaches display a marked capacity for structural insight and binding efficiency in aptamer design. Thereby, establishing a computational basis that supports the development of integrated, adaptive biosensing frameworks.

#### 3.3.4. Advanced Computational Frameworks for Multi-Target Biosensor Optimization

Adaptive computational frameworks extend the capabilities of biosensors by enabling multi-target detection. These frameworks employ multi-objective optimization techniques, such as ensemble learning, genetic algorithms, and reinforcement learning, to balance conflicting criteria, including sensitivity, specificity, and signal-to-noise ratio. In a recent review, the authors highlighted how adaptive frameworks leveraging ML and molecular modeling are able to generate high-affinity aptamers for multi-target detection, leading to significant enhancements in diagnostic accuracy [164].

New developments have demonstrated how adaptive frameworks can integrate ML with multiphoton effects to enhance biosensor sensitivity. Arano-Martinez et al. (2022) described how non-linear optical effects, when combined with ML, can significantly improve the detection capabilities of optical biosensors, allowing them to identify complex biological targets with high precision [165]. This approach not only enhances sensitivity but also provides a robust method for identifying low-dimensional agents, such as viral particles or rare biomarkers, in complex samples. Along similar lines, Raji et al. (2022) showed that ML-augmented biosensors can be used for the detection, stratification, and classification of biological cells in heterogeneous samples [166]. This work highlights how leveraging ML algorithms, such as SVM and neural networks, can automate biosensor development and lead to real-time, label-free classification of cell types, providing valuable diagnostic insights in clinical settings.

Moreover, an evolutionary computational intelligence framework for multi-target detection, optimizing sensor selection in multi-sensor systems was recently introduced. By employing a binary constrained evolutionary multi-objective algorithm that dynamically selects a subset of sensors at each time step, the model can significantly improve tracking accuracy while minimizing resource utilization [167]. Although the applied algorithm focused on multi-sensor systems, the underlying optimization principles are directly applicable to multi-target detection in biosensors. This showcases how adaptive computational frameworks, powered by ML and optimization algorithms, can fundamentally transform multi-target detection in biosensors, and enhance diagnostic precision and sensor performance.

Underpinning modern biosensor development, computational approaches enable rapid, precise, and scalable design. Tools such as ML, molecular simulations, and structural prediction optimize aptamer affinity, specificity, and stability while minimizing experimental trial-and-error. As the results indicate, these frameworks can accelerate prototyping and support the creation of multiplexed, high-performance diagnostic platforms.

### 3.4. Strategies for Improving Sensor Selectivity, Sensitivity and Signal Transduction

The sensitivity and signal transduction efficiency of aptamer-based biosensors are pivotal determinants of their analytical performance, particularly in applications requiring the detection of trace-level analytes. These functional parameters directly influence the sensor’s diagnostic utility, especially in complex biological and environmental matrices where low target concentrations and background interference are common challenges. Enhancing signal output while maintaining or improving specificity has thus become a central objective in aptasensor engineering.

A multitude of strategies have emerged to address this goal, beginning with general signal amplification methodologies that boost the detectability of binding events through chemical, physical, or molecular interventions. Among the most versatile and programmable approaches are nucleic acid-based amplification schemes, such as rolling circle amplification (RCA) and hybridization chain reactions (HCR), which exploit the replicative properties of oligonucleotide sequences to exponentially enhance signal output [168,169,170,171]. The aforementioned systems offer high target-to-signal conversion efficiency and are particularly amenable to integration with aptamer recognition elements. Another powerful avenue of innovation lies in the incorporation of nanomaterials. Metallic nanoparticles, carbon-based structures, and hybrid nanocomposites have demonstrated exceptional capabilities as both signal transducers and enhancers. Their tunable physicochemical properties facilitate electron transfer, fluorescence resonance energy transfer, and catalytic activity, and can therefore significantly augment detection sensitivity [172,173,174,175].

Enzymatic strategies also continue to play a crucial role, with recent advances in artificial enzymes and nanozymes offering enhanced stability and catalytic efficiency under diverse assay conditions. Furthermore, enzyme cascade reactions have been strategically assembled to generate amplified output through sequential reaction pathways [176]. Although enzyme-linked aptamer assays remain the gold standard for coupling molecular recognition with enzymatic signal transduction to detect a wide range of analytes from glycoproteins to viruses [177,178].

To increase binding precision and reduce false positives, dual-aptamer systems have been developed, wherein two distinct aptamers bind to different epitopes [179], synergistically improving both sensitivity and selectivity. Complementing these designs are structure-switching aptamers, engineered to undergo conformational changes upon target binding, thereby modulating the signal in a dynamic and controllable manner [180]. These molecular switches enable real-time monitoring and provide an additional layer of specificity for sensor operation. Integration of such strategies offers a multidimensional approach to optimizing biosensor performance. By combining amplification mechanisms with molecular engineering and material science, the boundaries of detection sensitivity and signal fidelity in aptamer-based sensing platforms continues to advance.

#### 3.4.1. Signal Amplification Techniques

Enhancing the sensitivity of aptasensors hinges on effective signal amplification strategies that convert low-abundance target recognition events into robust, quantifiable outputs. Amplification systems are indispensable for detecting analytes at femtomolar (fM) concentrations, particularly in complex biological samples where signal dilution and background noise often impede detection performance. Strategies like RCA, HCR, and catalytic hairpin assembly (CHA) leverage isothermal conditions to exponentially generate signal elements upon target recognition, bypassing the need for thermal cycling and in doing so offer compatibility with point-of-care and portable diagnostic platforms. RCA, for instance, involves the polymerase-mediated extension of a circular DNA template, resulting in long single-stranded DNA with repetitive sequences that can serve as scaffolds for signal probes. Electrochemical aptasensors utilizing RCA have demonstrated marked improvements in sensitivity, pushing detection limits to the fM range [181]. Combining RCA with techniques like CRISPR/Cas12a has been shown to be particularly effective at enhancing sensitivity. Qing et al. (2021) created a joint RCA-CRISPR/Cas12a system, which when employed in conjunction with an electrochemical platform enabled fM detection (1.26 fM) of thrombin, establishing it as an effective method of protein biomarker detection [182].

HCR and CHA further expand the signal without enzymatic involvement, enhancing system simplicity and stability. HCR initiates a cascade of hybridization events between two or more hairpin DNA structures upon target binding, while CHA employs catalytic strand displacement between engineered hairpins to continuously recycle the target and amplify signal generation. These enzyme-free strategies are particularly advantageous for resource-limited settings due to their low cost and reduced operational complexity [183].

Recent studies have increasingly explored combinatorial approaches that merge different amplification mechanisms to synergize their benefits. For example, integrating RCA with HCR and exponential isothermal amplification reaction (EXPAR) creates a novel amplification strategy, which as Zhang et al. (2025) showed enabled the ultrasensitive fluorescent detection of miRNA-21, ranging from 200 fM to 200 nanomolar (nM) [184]. Combining HCR with enzyme-assisted amplification presents another innovative approach to amplifying signal detection. As demonstrated in recent work, incorporating Klenow (3′→5′ exo-) and nicking endonuclease (Nb.BbvCI) enzymes with HCR creates large quantities of extended DNA (EXTDNA). In turn, harnessing EXTDNA facilitates the unfolding of hairpin structures immobilized on gold electrodes, which resulted in a highly sensitive electrochemical biosensor capable of detecting circulating tumor DNA (ctDNA) at a limit of 2.3 fM [185].

Another method within the combinatorial toolkit involves utilizing RCA to produce long and tandem DNA repeats and linking those to loop-mediated isothermal amplification (LAMP). Integration of LAMP with RCA (i.e., RCA-LAMP) produces a rapid and ultrasensitive method for microRNA detection at attomolar (aM) concentrations [186], illustrating it is an effective strategy for genetic biomarker detection. Such hybrid systems can jointly multiply the signal output and provide spatial and temporal control over amplification kinetics; thus, offering a versatile platform adaptable to various detection modalities.

#### 3.4.2. Nanomaterial-Assisted Signal Enhancement

Nanomaterials have emerged as pivotal components in enhancing the sensitivity and signal transduction efficiency of aptamer-based biosensors. Their unique physicochemical properties such as high surface area, superior electrical conductivity, and catalytic activity facilitate improved biomolecule immobilization and electron transfer, leading to amplified detection signals.

Gold nanoparticles (AuNPs) are extensively utilized due to their excellent biocompatibility and ability to facilitate electron transfer. Most notably, electrochemical immunosensors incorporating AuNPs exhibit marked improvements in sensitivity and detection thresholds across diverse use cases [187]. Similarly, carbon-based nanomaterials like graphene and carbon nanotubes (CNTs) offer high conductivity and surface area, enhancing the performance of biosensors. As a case in point, graphene-based E-AB have shown marked improvements in signal output due to graphene’s high conductivity, broad biocompatibility, and better surface modification capacity [188].

The combination of enzymes with nanomaterials has led to the development of hybrid biosensors with superior performance. These nanomaterials provide a conducive environment for enzyme immobilization, preserve enzymatic activity, and facilitate efficient electron transfer. Integrating horseradish peroxidase (HRP) with graphene oxide or graphene QDs can enhance electrochemical responses in biosensors, enabling the detection of analytes such as glutathione and hydrogen peroxide down to nM concentrations [189,190].

Moreover, nanocomposites (i.e., Fe_3_O_4_/CeO_2_@Au) offer a high surface area for biomolecule immobilization and facilitate electron transfer, improving biosensor performance through elevated signal strength. Significant advancements in biosensor specificity and sensitivity were demonstrated by Liu et al. (2018) when Fe_3_O_4_/CeO_2_@Au was utilized in conjunction with CHA [191]. Their work demonstrated improved detection capabilities for microRNA with a linear range of 1 fM to 1 nM, and a detection limit of 0.33 fM. This indicates that the strategic incorporation of nanomaterials into aptamer-based biosensors significantly enhances their sensitivity and signal transduction capabilities. By leveraging the unique properties of nanomaterials such as AuNPs, graphene, CNTs, and related nanocomposites, biosensors can be developed with improved performance metrics that facilitate the detection of low-abundance analytes.

#### 3.4.3. Enzyme-Mediated Amplification

Enzymes are integral to biosensor technology, offering catalytic capabilities that convert biochemical interactions into measurable signals. Traditional enzyme-linked immunosorbent assays (ELISAs) utilize enzymes like HRP and alkaline phosphatase (ALP) to amplify detection signals. Recent advancements have introduced innovative strategies to enhance enzyme-mediated signal amplification in biosensors. One such strategy is the use of nanozymes, artificial enzymes with peroxidase-like activity, which have emerged as adaptable, stable and cost-effective alternatives to natural enzymes. A representative example is iron-coordinated L-lysine-based nanozymes that have demonstrated effective catalytic activity for hydrogen peroxide and glucose detection in a hydrogel colorimetric biosensor [192]. The effective application of nanozymes to augment signal amplification in biosensor platforms further spans electrochemical, chemiluminescent, and fluorometric, to name a few [193].

Enzyme cascades, involving sequential enzymatic reactions, have been employed to amplify biosensor signals significantly. A notable example is the coupling of glucose oxidase (GOx) with HRP, which enhances the electrochemical response in glucose biosensors [194]. This application is best exemplified in the bienzymatic cascade catalysis of GOx and HRP in the DNA biomarker detection of Alzheimer’s disease specific sequences (aDNA). Gao et al. (2023) showed that when this enzyme cascade was coupled with electrochemical detection that it not only created a renewable biosensor but that it also possessed ultrahigh sensitivity, allowing for detection of aDNA at 0.22 fM [195]. As the research indicates, integration of enzyme-mediated amplification methods can be harnessed to address limitations inherent in single-method approaches, leading to more robust and versatile biosensing platforms.

While further exploration of enzyme-mediated amplification is beyond the scope of this review, it does warrant mention here for its value in illustrating another combinatorial technique that can be merged with biosensing to enhance signal amplification. For a more thorough overview of the application of nanozymes in personalized biosensors, see the review by Kurup & Ahmed (2023) [193].

#### 3.4.4. Dual-Aptamer Systems

Dual-aptamer systems have emerged as a sophisticated strategy in biosensor design, leveraging two aptamers that bind to distinct epitopes on a single target molecule. This dual recognition enhances both specificity and sensitivity, making these systems particularly valuable in complex biological environments. By targeting separate sites on an analyte, dual-aptamer configurations reduce the likelihood of cross-reactivity and false positives. This bivalent binding mechanism ensures that only the presence of the specific target triggers a detectable signal. This concept is well demonstrated in a dual-aptamer-based fluorescent biosensor that exhibited elevated sensitivity and specificity in detecting cancer-derived extracellular vesicles (EVs). The effectiveness of this approach is showcased by the functionalization of one aptamer to jointly detect CD63 and PKT7 while the other aptamer triggered RCA for signal amplification [196]. Such dual-aptamer strategies underscore a powerful paradigm for high-fidelity detection in next-generation biosensing platforms.

The versatility of dual-aptamer systems extends to various detection modalities, including electrochemical, fluorescent, and colorimetric assays. In electrochemical sensors, one aptamer can be immobilized on the electrode surface, while the second, labeled with a signaling molecule, facilitates signal generation upon target binding. Building on this approach, Guo et al. (2024) developed a biosensor that employed Au@Pd-functionalized aptamers to enable simultaneous electrochemical and colorimetric detection of lead—Pb(II), exploiting both differential pulse voltammetry and nanozyme-mediated TMB oxidation to reach a detection limit as low as 0.4 nmol L^−1^ [197]. Likewise, a dual-signal aptamer-based assay integrating CdTe quantum dot-functionalized magnetic nanoparticles and COF-Au nanozymes, enabled both FRET-regulated fluorescent quenching and tetramethylbenzidine (TMB) oxidation for colorimetric readouts [198]. These dual-mode detection schemes illustrate the analytical versatility and sensitivity achievable through aptamer-functionalized nanohybrids. Extending this concept to clinical diagnostics, a recent study introduced a dual-mode RNA-splitting aptamer biosensor that simultaneously employed colorimetric and fluorescent readouts to detect the HIV Tat peptide. By using carboxyfluorescein-labeled aptamers and gold nanoparticles, this system achieved signal transduction via FRET and nanoparticle aggregation that increased detection limits in human serum to 0.28 nM [199].

The increased specificity of dual-aptamer systems makes them particularly well-suited for detecting targets in complex biological matrices such as blood, serum, or food samples. This capability is crucial in clinical diagnostics and environmental monitoring, where the presence of interfering substances can compromise assay fidelity. Demonstrating this, Ou et al. (2019) [200] developed a dual-aptamer sandwich-type electrochemical aptasensor for sensitive and selective detection of the breast cancer biomarker Human Epidermal Growth Factor Receptor 2 (HER2). One aptamer was immobilized on a gold electrode via a tetrahedral DNA nanostructure to capture HER2, while the second was linked to a multifunctional nanoprobe comprising flower-like Mn_3_O_4_/Pd@Pt nanozymes and HRP to catalyze signal generation. Ultimately, formation of a dendritic nanostructure led to marked signal amplification, enabling a detection limit as low as 0.08 ng·mL^−1^ [200]. This method further underscores the value of combining multiple amplification strategies for more accurate biomarker quantification.

Building on the improved detection capacities afforded by dual-aptamer specificity, the cooperative binding of two aptamers also facilitates signal amplification, which is of particular importance when analytes are present at low concentrations. Illustrating the effectiveness of this approach, a dual-aptamer electrochemical sandwich biosensor was designed for the sensitive detection of Michigan Cancer Foundation-7 (MCF-7) breast cancer cells and the Mucin 1 (MUC1) tumor marker. In this system, a MUC1-specific aptamer was immobilized on a multiwall carbon nanotube/poly(glutamic acid)-modified electrode, while a secondary aptamer conjugated to silver nanoparticles enabled amplified signal generation via anodic stripping voltammetry, which achieved a detection limit as low as 25 cells·mL^−1^ [201]. Expanding upon this type of signal enhancement strategy, a multielectrode array (MEA)-based biosensor using electrodeposited 3D nanostructures to immobilize aptamers specific to both ATP and amyloid-β oligomers (AβO) enabled the simultaneous, and highly specific electrochemical detection of both biomarkers, with limits of detection of 0.01 nM and 1 pM, respectively [202]. The clinical utility of this dual-aptamer configuration for early Alzheimer’s disease diagnosis showcases how spatially engineered sensor platforms can enable multiplexed, ultrasensitive biomarker detection. As evinced across multiple applications, from heavy metal and viral biomarker detection to cancer and neurodegenerative diagnostics, dual-aptamer strategies significantly strengthen the analytical performance and multiplexing capacity of biosensor frameworks.

#### 3.4.5. Structure-Switching Aptamers

Structure-switching aptamers (SSAs) have emerged as a pivotal innovation in biosensor technology, offering enhanced sensitivity, specificity, and real-time detection capabilities. These aptamers undergo conformational changes upon target binding, directly translating molecular recognition events into measurable signals, thereby eliminating the need for external reagents.

Some of the conceptual groundwork for structure-switching aptamer design was laid by Feagin et al. (2018) [203], who examined multiple strategies for incorporating structure-switching functionality into existing aptamers. Through thermodynamic analyses, they identified critical variables influencing switch efficiency and signal fidelity, and emphasized the value of embedding this functionality during the aptamer selection process to streamline biosensor development [203].

In a recent paper, Yoshikawa et al. (2023) [204] introduced a massively parallel screening strategy that complements these methodological advancements by enabling the transformation of nearly any aptamer into a structure-switching molecular switch without requiring prior structural knowledge of the aptamer [204]. Unlike conventional in silico modeling, often limited by the inability to accurately predict three-dimensional conformations or non-canonical base pairing, this strategy bypasses these constraints by experimentally screening a vast number of variants. Results showed an approximately 30-fold greater sensitivity than other DNA-based alternatives [61]. Application of this approach offers a scalable and generalizable method for producing target-responsive biosensors with high sensitivity and rapid response kinetics.

A notable advancement in computational aptamer design was demonstrated in 2022 through a smart SELEX framework that integrated ML, bioinformatics tools, and MD simulations to engineer RNA aptamers with target-responsive conformational changes. These aptamers were incorporated into an electrochemical sensor for detecting ammonium, showcasing strong selectivity and sensitivity based on structural adaptability [61]. Expanding on this trajectory, DeepAptamer, a hybrid CNN–BiLSTM DL model trained on SELEX-seq data to predict high-affinity aptamers from early selection rounds was recently introduced [67]. By identifying critical binding motifs and bypassing enrichment biases, this computational tool improves aptamer recovery and facilitates the discovery of candidates with structure-switching potential.

Extending the trend toward computational innovation in aptamer engineering, a Python-based in silico pipeline was developed to convert stable-conformation aptamers—typically selected via conventional SELEX—into structure-switching variants suitable for biosensing [205]. By integrating binding motif information with secondary structure data, the algorithm generated three ssDNA libraries, which facilitated the identification of a novel aptamer (MSA-Apt-16) with high affinity (*K_D_* = 7.6 nM) [205], underpinning the potential of computational approaches in designing efficient structure-switching aptamers for diagnostic applications.

SSAs mark a key advancement in biosensor technology, linking target recognition to signal generation via conformational switching. Methods like parallel screening have scaled the design of switchable aptamers, while ML, deep neural networks, and in silico modeling have accelerated the discovery of high-affinity, responsive sequences. Such progress illustrates how the convergence of experimental and computational innovations is driving the evolution of SSAs toward more adaptable and high-performance diagnostic tools.

## 4. Aptasensor as Point-of-Care Diagnostic Solution and Commercialization

With ongoing research efforts focused on improving the aptamer selection technologies and the biosensor architectures, numerous aptasensors enabling sensitive and specific detection have been reported every year. Nonetheless, successful translation from laboratory prototypes into commercialized diagnostic products remains scarce, particularly for Point-of-Care Testing (POCT). As initially described by the WHO in 2004 and recently redefined by researchers, POCT should exhibit a set of characteristics abbreviated as REASSURED [206,207]. Table 3 below describes the nine criteria.

It is evident that developing an assay with high sensitivity and specificity alone is insufficient for deployment at the POC level. The other criteria listed in Table 3 are equally important. For example, a POCT should provide rapid diagnostic results to delay disease progression by enabling timely treatment, ultimately mitigating mortality. The assay ideally should be affordable and accessible to end-users in need, regardless of their financial and geographical status. The test should also be easy to perform, from specimen collection to result interpretation. Altogether, an ideal diagnostic assay exhibiting all of the REASSURED characteristics would be most likely to improve global healthcare by decentralizing disease diagnosis from clinical settings to the Point-of-Care level [208,209,210,211].

Several aptamer-based biosensing platforms have been reported to accurately and specifically detect analytes using sophisticated devices such as SERS, Biolayer Interferometry (BLI) and optical fiber biosensors [212,213,214,215,216,217]. However, most of the aptasensors listed are intended for clinical or research applications, often featuring prolonged detection protocols, the use of specialized equipment and the necessity of result interpretation by professionals. The following section explicitly evaluates recent developments in aptamer-based biosensors based on the POCT criteria, highlighting examples of LFA and E-AB with potential for POCT commercialization. We also discuss the challenges ahead.

### 4.1. Lateral Flow Assay

#### 4.1.1. Principle of LFA

**Figure 4:**
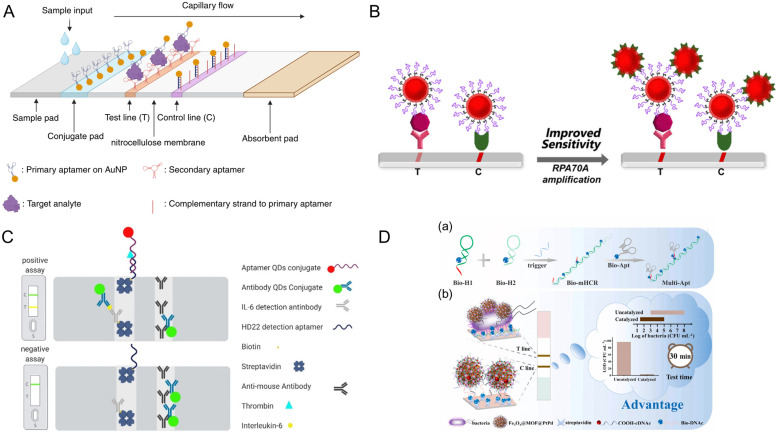
Aptamer-based lateral flow assay. (**A**) Schematic illustration of a typical sandwich binding aptamer LFA. Created in BioRender (https://BioRender.com/knho7e3 accessed on 8 May 2025). (**B**) Schematic figure of aptamer-mediated lateral flow detection of influenza A, B and COVID-19 [223]. The test line was printed with specific antibodies while the aptamers were immobilized on AuNPs for sandwich binding. Replication protein A 70 kDa subunit domain (RPA70A) coated AuNPs recognized unbound aptamers and clustered onto the T and C lines, improving the colorimetric signal. Copyright © 2024, American Chemical Society. (**C**) Schematic diagram of the aptamer and antibody-quantum dot sandwich LFA for thrombin and interleukin−6 [220]. The fluorescent red quantum dot conjugated with the thrombin binding aptamer while the green quantum dot conjugated with interleukin−6 antibody. Copyright © 2020 Elsevier B.V. All rights reserved. (**D**) Schematic of multivalent aptamer magnetic nanozyme (MA-MN) LFA for pathogen detection [224]. (**a**) Synthesis of hybridization chain reaction (HCR) multiple aptamer construct. (**b**) Sandwich binding of bacteria by vancomycin-modified nanozyme termed Fe_3_O_4_@MOF@PtPd and multi-aptamer construct captured by the streptavidin printed test line. Catalytical conversion of substrate 3,3′-diaminobenzidine (DAB) improves both the detection time and detection range. Copyright © 2024 Published by Elsevier B.V.

LFA was first proposed in 1960 by biophysicist Rosalyn S. Yalow and physician Solomon A. Berson to detect plasma insulin [218]. Since then, the technology has expanded rapidly to detect critical biomarkers ranging from hormones, proteins, and viral particles to whole cells [219,220,221,222]. The LFA is a paper-based device assembled from four major components (Figure 4A). Biofluid is first introduced onto the sample pad, which is saturated with buffer solution and detergents to facilitate homogenous molecular solvation and unidirectional flow into the adjacent conjugate pad through capillary action. The conjugate pad typically contains dried aptamer immobilized gold nanoparticles (Apt-AuNPs). As the sample flows through, target biomolecules bind to the Apt-AuNPs, and the complexes continue to flow into the nitrocellulose membrane. The membrane consists of two parts: a control line printed with DNA probe complementary to the primary aptamer sequence to capture the Apt-AuNPs regardless of the presence of target molecule, and a test line usually printed with a secondary aptamer to capture only the binding complexes through sandwich binding. Successful retention of AuNPs on either line leads to the development of a visible band due to the nanoparticle clustering effect. This is known as the sandwich LFA where the line intensity is directly proportional to target concentration. Finally, the sample passes through the membrane and reaches the absorbent pad to soak up excessive solution and complete the test.

#### 4.1.2. Point-of-Care Applications of Aptamer Sandwich LFA

Lateral flow is regarded as a promising POCT platform due to its alignment with numerous characteristics outlined in the REASSURED framework. For example, LFA shares exceptional affordability compared to any other platform. This is attributed to its simple design comprising only a few components to assemble a functional assay. In addition, miniaturization significantly reduces production costs through high-volume manufacturing. Finally, the raw materials involved are relatively inexpensive. This includes not only the nitrocellulose membrane and AuNPs, but also the use of aptamer over traditional antibodies. Aptamers are known for their low production cost from solid phase oligonucleotide synthesis compared to antibodies which are produced in vivo and the necessity to undergo rigorous purification from cell lysate before uses [225]. Therefore, aptamer-based LFAs represent a cost effective POCT solution in resource-poor areas.

Apart from affordability, LFAs offer rapid diagnostic turnover time and intuitive visual interpretation requiring no user expertise. These attributes are particularly important when it comes to community-level infectious disease control. A prototype described by Le et al. (2017) [226] outlined an antibody-aptamer sandwich LFA targeting influenza A H3N2 (A/Panama/2007/99). In contrast to the typical aptamer sandwich construct, this model featured co-deposition of biotinylated aptamers and antibody coated AuNPs on the conjugate pad. In the presence of target viral proteins, sandwich binding occurred between the antibodies and the aptamers. The binding complexes were captured by streptavidin test line to develop signal observable by naked eye. The assay allows rapid detection in 15 min with a limit of detection (LoD) at 2 × 10^6^ virus particles, matching clinical viral loads in respiratory samples. This suggests practical applications for voluntary self-testing against seasonal flu. Nonetheless, it should be noted that its efficacy has neither been proven in spiked nor real patient samples.

Multiple aptamer-based LFA have been reported targeting SARS-CoV-2 in response to recent pandemic outbreaks. For instance, Derin et al. (2024) developed a flow test against SARS-CoV-2 Spike (S) protein selecting from six aptamer-aptamer pairs [227]. Among these pairs, sandwich binding by the identical aptamer RBD-4C proved most effective [228], developing a visible test line signal in 5 min. The assay demonstrated 100% clinical sensitivity and 93.3% specificity agreement with RT-qPCR results using nasopharyngeal and oropharyngeal swab from confirmed COVID-19 patients. While the study did not verify the detection limit of the S protein, it shows that aptamer mediated LFA may achieve diagnostic accuracy comparable to traditional PCR methods. Similarly, Yang et al. (2022) developed another sandwich LFA against the S protein by the aptamer pair SNAP1 and SNAP4 pair [229,230]. It achieved a LoD at 10^6^ copies/mL, considered a high viral load sample. The clinical utility was verified via spiked healthy nasal swab samples. However, the assay takes one hour to complete due to the lack of a conjugate pad for rapid binding. Further optimization on the strip design is likely to reduce the assay time for better POC applications. Universal aptamers against S proteins from multiple COVID variants were selected by Kim et al. (2025) to address the potential loss of specificity resulting from continuous viral mutations [231]. The study developed a new aptamer pair AM086-1 and AM016 for sandwich LFA design. A highlight from the study was the successful recognition of live SARS-CoV-2 wild type, delta and omicron strains with visual LoDs of 1.4 × 10^6^, 1.8 × 10^5^ and 3.6 × 10^5^ TCID_50_/mL respectively in 20 min. Collectively, these examples demonstrate the great potential of aptamer-based LFAs for POC-level infectious disease diagnosis.

#### 4.1.3. Recent Advancement in Developing Ultrasensitive Aptamer-Based LFA

While proof-of-concept studies have demonstrated the efficacy of aptamer-based LFAs for infectious disease control, they have not yet surpassed traditional assays to become the primary diagnostic method. This is likely because the cost effectiveness, speed and ease of use of LFAs have not fully compensated for the sensitivity discrepancy compared to existing technologies, especially for detecting trace biomarkers in the early-stage disease progression. In this section, we summarize recent research addressing the sensitivity gap and strategies to promote future commercialization.

##### Secondary AuNPs Clustering

The colorimetric signal intensity developed on the test line is critically influenced by the nanoparticles clustering density. Kim et al. (2024) reported a signal amplification strategy by introducing an extra conjugate pad infiltrated with the RPA70A coated gold nanoparticles (RPA70A-AuNPs) to the traditional sandwich design [223]. As shown in Figure 4B, RPA is a single-stranded DNA binding protein, allowing RPA70A-AuNPs to bind to Apt-AuNPs. This increased the clustering concentration of AuNPs on the test line and significantly improved LoDs of influenza A, B and COVID-19 by 4-folds to 2.89 pg/mL, which is 200-fold lower than a commercial antibody LFA. The strategy successfully enhanced diagnostic sensitivity without compromising the assay simplicity and rapidity (i.e., 20 min signal development).

##### Quantum Dots (QDs)

Replacing AuNPs with fluorescence QDs is another strategy to enhance signaling efficiency because of their high fluorescence yield and photostability [232]. Li et al. (2025) developed a multiple aptamer recognition-based quantum dot lateral flow immunoassay (MARQ-LFIA) against SARS-CoV-2 nucleocapsid (N) protein [233]. They substituted Apt-AuNPs with Apt-QDs and forming sandwich complexes captured at antibody-immobilized test lines. This approach reached a LoD for the N protein at 1.4 pg/mL, among the lowest reported for N protein LFAs. Mahmoud et al. (2021) conjugated thrombin aptamer and interlukin-6 (IL-6) antibody separately onto red- and green-emitting fluorescence QDs, respectively [220]. Simultaneous duplex detection was achieved on the same test line capturing both targets (Figure 4C). The estimated LoDs of thrombin and IL-6 were 100 pM and 3 nM, respectively, which are similar to other reported aptamer LFA models [234,235,236]. While fluorescence readouts traditionally require specialized equipment, limiting POC use, advancements in smartphone imaging and analysis technologies show promise for transitioning to portable fluorescence-based LFAs with superior analytical sensitivity [220,237,238,239].

##### Nanozyme

Lastly, signal intensity can be amplified using nanozyme-mediated catalytic reactions to generate visible redox products on the test line. Sarathkumar et al. (2025) leveraged the intrinsic peroxidase-mimic activity of AuNPs to enhance the colorimetric signal [240]. Following the standard sandwich binding configuration using a pair of aptamers targeting the COVID S protein, the Apt-AuNPs catalyzed the conversion of para-phenylenediamine (PPD) into a dark brown product Bandrowski’s base (BB), creating a strong color contrast against the nitrocellulose membrane. As a result, it extended the visible detection limit from 4 ng/mL to 200 pg/mL, with a statistical LoD to 168 pg/mL. The signal amplification step is rapid (<5 min), maintaining the assay’s speed comparable to traditional LFAs. Similarly, Li et al. (2024) substituted AuNPs to a metallic nanozyme termed Fe_3_O_4_@MOF@PtPd to catalyze the DAB into brown precipitates (Figure 4D) [224]. The proposed sensor detected *Staphylococcus aureus* by sandwich binding between vancomycin modified nanozymes and biotinylated DNA aptamers against the bacteria. The complexes were captured by the streptavidin test line, reaching a LoD of 97 CFU/mL and 2 CFU/mL before and after signal amplification, respectively. Nanozyme catalysis is fast (i.e., within 5 min) and compatible with traditional AuNPs or AuNP alloy systems [240,241,242,243]. Therefore, it can be readily adopted to existing designs for enhanced sensitivity.

In short, LFA has been a popular technique for developing POCT. Aptamer integration enables cost-effective, high-throughput manufacturing. However, LFAs generally lack sufficient clinical sensitivity for early-stage disease diagnosis. Practical solutions and examples to improve the detection limit have been discussed, aiming to empower LFAs as high-sensitivity and quantitative diagnostic tools.

### 4.2. Electrochemical Aptamer Biosensor (E-AB)

Electrochemical sensors have been commercially deployed in various fields, with continuous glucose monitoring (CGM) being a well-known example utilizing the enzyme glucose oxidase for binding and signal transduction [244]. The significance of CGM extends beyond its sensitivity, offering user friendliness through wearable electronics that enable real-time glucose monitoring. However, bioreceptors like enzymes have a limited target range due to substrate specificity, while antibodies are only effective for immunogenic targets and incur high production cost [245]. Consequently, there is a growing interest in developing E-ABs, especially for POC level applications because of their rapid and reversible response, device miniaturization potential, high sensitivity and unique real-time monitoring capabilities [246,247,248].

With the exception of transistor designs, typical E-ABs are configurated in a classic three-electrode system consisting of a counter electrode, a reference electrode and a working electrode with DNA aptamers immobilized on the surface. When targets bind to the aptamers, changes in surface electrochemical properties are interrogated by a potentiostat and transduced into readable signals. This section discusses recent developments in E-ABs focusing on differential interrogation techniques and highlighting their advantages and disadvantages in relation to POCT commercialization.

#### 4.2.1. Amperometric E-ABs

Amperometry biosensing measures electrical current over time under constant applied voltage. Similarly to nanozyme-enhanced LFAs, amperometry often relies on redox species generation from enzymatic reactions. Several studies have detailed the potential diagnostic applications of amperometric E-ABs [249,250,251,252]. Despite high sensitivity, the requirement for multiple washing and substrate feeding steps makes it better suited for clinical and laboratory settings than POCT.

Recently, researchers have developed a new chronoamperometric sensing mechanism called molecular pendulum. First reported as an antibody-based biosensor for troponin detection [253], it was later adapted to an aptamer-pendulum format for B-type natriuretic peptide (BNP) sensing in whole blood [254]. It consists of a rigid double-stranded DNA helix in which one strand is extended by an aptamer and the complementary strand is labeled with a ferrocene redox reporter. A positive voltage applied to the working electrode attracts the negatively charged DNA onto the electrode surface, enabling faster electron transfer due to the redox reporter’s proximity. Chronoamperometric interrogation measures the time required to reach hydrodynamic equilibrium. Target binding increases the molecular size and the drag force experienced by the aptamer-pendulum in a concentration-dependent manner. Thus, the analytes can be quantified by the difference in equilibration time between bound and unbound states. The sensor achieved a remarkable LoD of 10 fg/mL in both PBS and whole blood within 30 min.

**Figure 5:**
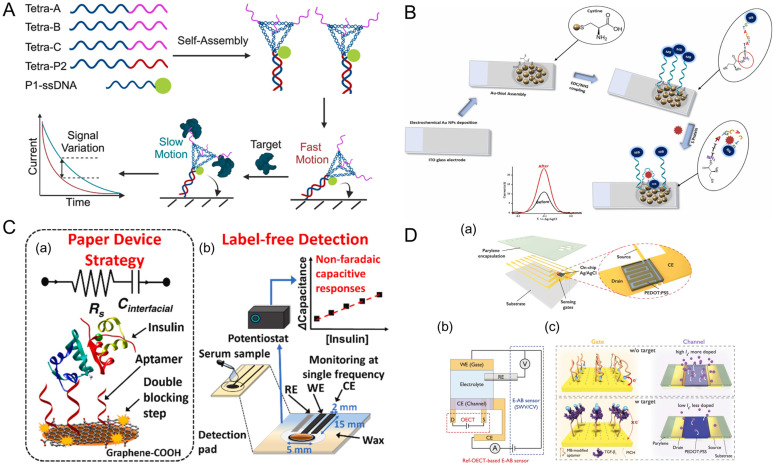
Aptamer-based electrochemical biosensors using amperometry, voltammetry, impedance spectroscopy and transistor interrogation techniques. (**A**) Schematic illustration of chronoamperometric continuous biosensing by tetrahedral DNA nanostructure pendulum design. The green dot linked with P1-ssDNA is the ferrocene (Fc) reporter. Figure reproduced from [255], licensed under CC BY 4.0. (**B**) Schematic representation of SARS-CoV−2 Spike protein detection by structural switching E-AB on an Indium Tin Oxide (ITO) electrode functionalized with AuNPs and cystine for methylene blue (MB)-modified aptamer immobilization. Copyright © 2023 Elsevier B.V. All rights reserved [257]. (**C**) Schematic illustration of non-faradaic E-AB for serum insulin biosensing [258]. (**a**) Graphene electrode surface biofunctionalization with amine-modified aptamer. Double blocking step refers to the sequential surface passivation by BSA and ethanolamine, (**b**) Design and dimension of the graphene electrode coupled with a potentiostat for capacitance measurement. Copyright © 2022 Published by Elsevier B.V. (**D**) Schematic of OECT-based E-AB for transforming growth factor beta 1 (TGF−β1) sensing. (**a**) Design of the OECT device. (**b**) Electric circuit for concurrent detection by OECT-based and standard square wave voltammetry (SWV) or CV-based E-AB. (**c**) Signal “OFF” electrochemical sensing of TGF−β1 in OECT-based E-AB leads to reduced transistor current modulation (less doped). Figure reproduced from [259], licensed under CC BY 4.0.

Expanding on this work, Wang et al. (2024) enhanced the pendulum design by incorporating DNA tetrahedron nanostructures (Figure 5A) or AuNPs to display multiple aptamers per helical anchor [255,256]. They successfully detected VEGF and thrombin in artificial urine with excellent femto- to picomolar LoDs. The aptamer-pendulum model provides a reagentless, continuous and drift-free electrochemical biosensing solution suitable for POC applications. However, very limited studies have validated its efficacy for biomolecules with diverse physiochemical properties (e.g., size and charge). Additionally, benchmarking requirements and calibration against background matrices complicated absolute quantification, potentially compromising reproducibility across devices.

#### 4.2.2. Voltametric E-ABs

Voltammetry interrogation measures electrical current as the applied potential varies. This technique is commonly used in structural switching aptasensor where aptamers are modified with redox reporters such as methylene blue (MB). Target binding induces aptamer conformation change, altering the distance of the reporter to the electrode surface, thereby affecting the electron transfer rate. Voltammetry scan at the redox potential reveals electrochemical current changes, with signal “ON” indicating an increase in current due to binding and signal “OFF” indicating the opposite. While redox labeling increases E-AB cost, it allows rapid, single-step and highly specific detection. Reversible target binding further facilitates sensor regeneration for continuous and real time in vivo molecular sensing [246,260].

Recent examples demonstrating potential POC applications of structural switching E-ABs include SARS-CoV-2 antigen detection. Siu et al. (2024) developed E-ABs against the N protein by square wave voltammetry (SWV) interrogation [261]. This method signifies rapid detection in 5 min, with real-time SWV scans every 15 s. The signal plateaued at the 10-min mark with decent sensitivity at low nanomolar LoD and detection compatibility in a salivary matrix for POCT deployment. Idili et al. (2021) was the first to report a structural switching E-AB for S protein detection within 15 min using SWV [262]. The prototype employed the aptamer, RBD-1C and showed high picomolar binding affinity (*K_D_*) against the target in fetal bovine serum (FBS) and 50% artificial saliva. Later, Curti et al. (2022) reported a similar folding-based E-AB for S protein sensing using another published aptamer, CoV2-6C3 [263]. Though the E-AB detection required 2 h of sample incubation, it achieved low nanomolar LoD in viral transport medium (VTM), a buffer frequently used for nasopharyngeal swab sampling. Lastly, Khan et al. (2024) characterized S protein detection using RBD-1C immobilized on ITO electrodes via EDC/NHS amine coupling (Figure 5B) [257]. By differential pulse voltammetry (DPV) interrogation, their E-AB achieved a LoD of 91 pM in human saliva and serum within 35 min.

##### Challenges and Potential Solutions for Structural Switching Voltametric E-AB Commercialization

Structural switching E-AB benefits from the reagentless, reversible and single-step detection design, substantially simplifying the detection protocol. It offers sufficient specificity because signal transduction is mediated by the aptamer’s conformational change upon binding to its cognate target. However, there remain a few unsolved challenges in the pursuit of commercialization.

A strong structural switching property is crucial for successful E-AB adaptation, in addition to high binding affinity. Yet, traditional evolution does not select for such characteristics, often yielding high-affinity sequences with suboptimal structural dynamics. Recent solutions can be categorized as pre-selection or post-selection modifications. Pre-selection modifications, such as designing a pre-defined structural library [264,265], fluorescence guided evolution of structural switching aptamers [264,266] or restriction enzyme mediation selection (RE-SELEX) [267] aim to evolve significant conformational switching properties from the starting SELEX libraries. One notable study employed RE-SELEX evolved aptamers to detect kanamycin A via target-induced strand displacement and catalytic specificity of the nucleases [267]. Post-selection modifications such as Nuclear Magnetic Resonance (NMR) guided sequence truncation [268] and pseudoknot sequence extension [269,270] retrofit existing aptamers with structural switching properties, specifically for the one-step E-AB model.

Aptamer monolayer stability remains a long-standing challenge affecting both in vitro and in vivo sensing accuracy. Biofouling and monolayer desorption have been identified as the primary causes of signal degradation over time [271]. In response, various solutions have been proposed. These include preservation of monolayers by optimizing the voltametric scanning parameters [272], drift correction using Kinetic Differential Measurement (KDM) [273] or dual-aptamer differential signaling [274] and alternative surface blocking chemicals to resist biofouling [275,276]. Though several studies have proposed methods to prolong E-AB stability in complex matrices, optimal antifouling solution requires case-by-case experimentation tailored to specific sample matrices and potential interferences.

Device calibration and benchmarking pose another key challenge for structural switching E-AB. Electrode surface area and microstructure play a crucial role in determining the absolute MB faradaic current intensity and aptamer packing density [277,278], both are essential for optimal sensor performance. However, maintaining consistent control of these parameters across sensor batches is difficult. Standardization through calibrating against analyte-free samples is thus necessary for reproducibility but impractical for large-scale fabrication. Consequently, several calibration-free measurement solutions have been proposed. The general concept is to introduce an internal standard where the reference signal remains unchanged in the presence of the target molecules. Such a standard can either be an alternative redox reporter located at a fixed distance from the electrode surface [279,280] or an additional measurement taken at “non-responsive” SWV frequencies [281,282]. These methods have demonstrated clinical success in complex matrices such as in vivo continuous sensing in mice [280]. Yet it is important to note that existing calibration-free E-AB studies primarily focus on small molecules or hormones, with limited exploration in efficacy for proteins, extracellular vesicles, or cells. A comprehensive investigation in this area would likely benefit the commercial application of structural switching E-ABs in diverse healthcare settings.

#### 4.2.3. Impedance E-ABs

Electrochemical impedance spectroscopy (EIS) measures electrical resistance from an alternating current (AC) circuit in an electrochemical system by applying a small amplitude AC voltage and analyzing the current response across a frequency range [283]. When combined with aptamers, impedance E-AB measures the change in interfacial properties when targets are captured on the electrode surface. EIS offers label-free detection and operates in two detection modes: (1) faradaic and (2) non-faradaic.

##### Faradaic EIS

Faradaic detection measures charge transfer resistance (*R_ct_*) using a pair of redox coupling species, usually ferri/ferrocyanide (i.e., [Fe(CN)_6_]^3−/4−^). As aptamers capture the targets, charge transfer efficiency reduces because of the steric hindrance and electrostatic repulsion (i.e., increase in *R_ct_*) [284]. The analyte concentration can be quantified from the *R_ct_* difference compared to a blank sample. Several studies have demonstrated the applications of aptamer faradaic EIS to detect key molecular markers such as VEGF [285], prostate cancer antigen 3 (PSA3) [286] and carcinoembryonic antigen (CEA) [287] with satisfactory clinical sensitivity and specificity. However, faradaic EIS has not yet proven to be an encouraging POC solution. Faradaic EIS requires additional steps to introduce the essential redox mediators for signal generation, unlike the single-step structural switching E-ABs. The pair of anionic redox species has been reported to etch and deteriorate gold electrode surface in phosphate buffer [288,289,290], leading to signal drift overtime. There are also inconsistencies in the literature. For example, Erdem et al. (2024) reported an increasing *R_ct_* signal detecting the SARS-CoV-2 S protein [291] but Abrego-Martinez et al. (2022) demonstrated a decreasing *R_ct_* response to the same target [292], which is explained by the attraction of anionic redox species to the positively charged proteins, facilitating rather than inhibiting charge transfer. Similar study has been reported to detect *Plasmodium falciparum* lactate dehydrogenase (PfLDH) where the response varies depending on the pH and the protein’s isoelectric point [293]. These findings raise concerns about the feasibility and reliability of faradaic EIS for detecting positively charged biomolecules under physiological conditions.

##### Non-Faradaic EIS

Non-faradaic EIS measures changes in electrical double layer capacitance (*C_dl_*) at the electrode surface. Target binding alters the dielectric properties of this layer without redox reactions. Therefore, it supports label-free, reagentless and possibly single-step detection. Niroula et al. (2022) reported a capacitance-based aptasensor for insulin [258]. The prototype was developed on a miniaturized paper-based device and verified in PBS-diluted serum, achieving a remarkable 1.5 pM LoD in 30 min (Figure 5C). Reduced sensitivity was reported when tested in undiluted serum, suggesting a potential biofouling issue for further optimization. Sánchez-Salcedo et al. (2023) reported another non-faradaic aptasensor for IL-6 [294]. They compared capacitance sensing by nanobody and aptamer coated gold arrays and realized IL-6 detection from 10 pg/mL to 10 ng/mL. An inevitable capacitance drift overtime can still be observed despite implementing rigorous blocking steps. This may suggest a common challenge for non-faradaic EIS, where biofouling and non-specific adsorption of biomolecules from complex matrices cause target-independent signal drift. Research into alternative blocking schemes or drift correction methods could advance this analytical application.

#### 4.2.4. Transistor E-ABs

E-ABs can be integrated into field-effect transistor (FET) or organic electrochemical transistor (OECT). Unlike the typical three-electrode setting, FETs and OECTs involve source, drain and gate electrodes with a transistor channel printed between the source and drain. While traditional E-ABs measure the changes in electrochemical surface properties at the aptamer functionalized working electrodes, transistor E-ABs measure the change in channel conductivity modulated by the gate electrode where aptamers are immobilized. A major difference between FET and OECT is the mechanism modulating the channel conductance, as explained by a previous review [295]. Briefly, FETs rely on electric field driven modulation, whereas OECTs operate through electrochemical doping via bulk ions injection into the channel. Both exhibit diverse advantages including a high signal amplification factor for trace biomarker sensing. Wang et al. (2022) reported an FET coupled with a molecular cantilever built on a DNA tetrahedron, achieving a remarkable LoD of COVID RNA at 0.02 copies/μL in undiluted saliva [296]. The FET combined with DNA aptamer successfully detected thrombin from undiluted serum with aM sensitivity, surpassing most of the classic E-ABs. Ji et al. (2023) developed an aptamer-OECT to amplify the electrochemical signal on-site for transforming growth factor beta 1 (TGF-β1) detection [259]. The aptamers were labeled with MB reporter and the binding of TGF-β1 drives the moiety further away from the gate electrode, reducing gate and channel currents (Figure 5D). The sensitivity of aptamer-OECT (290 μA/dec) was 4 orders of magnitude higher than the standard SWV E-AB setting (84 nA/dec). Another noticeable advantage is the fabrication of miniaturized and flexible OECT [297,298], which is applicable as a wearable electronic skin patch. Advantages aside, the lack of standardized data analysis methods in the field remains an issue. While some researchers measured the transistor current change (∆*_IDS_*) [299,300], others measured the gate potential shift (∆*G*) [301,302]. Establishing unified signal processing methods to optimize the signal gain is crucial for translating transistor-based E-ABs to POC applications.

In summary, E-ABs stand out as a promising solution for POCT due to their rapidity, cost-effectiveness, scalability, sensitivity, and specificity. While structure-switching E-ABs offer facile assay design and simplicity for real-time monitoring using voltametric methods, advancements in E-ABs based on other interrogation techniques provide propitious alternatives for miniaturized, aptamer-based electrochemical diagnostic solutions. Beyond differentiating various electrochemical interrogation techniques, emerging engineering technologies can be integrated into existing E-AB models. For instance, recent advances in soft electronics combined with microneedle technologies have facilitated the creation of wearable biosensors for real-time and continuous molecular monitoring with enhanced user comfort and portability [303,304]. These innovations are showcased by biotech companies such as Nutromics and Biolinq. Similarly, the integration of miniaturized potentiostats from companies such as PalmSens and MicruX Technologies has enabled smartphone applications, offering intuitive interfaces, remote data transmission and decentralized diagnostic capabilities for a broad range of health conditions. Finally, microarray fabrication technologies enable co-deposition of multiple DNA aptamers onto a biosensor device [305], facilitating simultaneous detection of multiple analytes through a single measurement at the point-of-care. Collectively, these emerging technologies hold great promise for refining the future of digital health.

## 5. Discussion and Perspectives

Aptamer-based biosensors have reached a stage where nominal sensitivity must be weighed against matrix compatibility, stability during operation, and deployability under point-of-care (POC) constraints. The REASSURED framework emphasizes that sensitivity and specificity, while necessary, are insufficient predictors of utility without parallel attention to robustness, time-to-result, user-friendliness, cost, and connectivity [206,207,208,209,210,211]. In practice, devices that perform credibly in blood, saliva, or urine do so because the recognition element is predisposed to switching and stable binding, the interface preserves conformation while resisting fouling, and the readout suppresses drift or precludes calibration steps that are impractical outside controlled laboratories.

Selection quality is the upstream determinant of downstream reliability. Modern selection formats like capture and CE variants, microfluidic partitioning, and Toggle-style strategies tighten stringency, reduce rounds and reagent burden, and can bias libraries toward aptamers that undergo target-induced conformational change [28,29,31,32,34,35,37,39,42]. That bias is consequential such that structure-switching candidates natively align with reagentless transduction and time-resolved monitoring, and they tolerate matrix variability better than binders optimized solely in buffered discovery conditions [246,255,262,265]. Data-driven pipelines extend this advantage. ML analysis of round-resolved sequencing, in conjunction with structure-aware modeling (secondary/tertiary prediction, docking/MD), accelerates identification of high-value sequences and reduces enrichment bias [60,61,63,64,67,70]. In practice, these tools raise the fraction of switch-competent, matrix-tolerant candidates entering device development, shorten iteration cycles, and help curb LoD inflation in complex fluids by prioritizing motifs that retain conformation under realistic ionic strength, pH, and interferent backgrounds.

Outside of ML pipelines, harnessing signal design allows for connection of selection and surface chemistry to specificity, dynamic range, and multiplexing. An exemplification of this is dual-aptamer “sandwich” architectures that have been shown to increase specificity via cooperative binding and facilitate enzymatic or nanozyme amplification. Along similar lines, multielectrode arrays extend this concept to parallel detection, with representative demonstrations achieving sub-nanomolar and picomolar limits for distinct analytes on the same platform [200,201,202]. Structure-switching aptamers unify recognition and transduction in reagentless formats that are compatible with one-step workflows and continuous monitoring. This facilitates the capacity to both select for switching and to convert fixed-conformation binders into switches at scale, thus enabling systematic exploration of switchable motifs alongside performance benchmarking [203,204,205].

Electrochemical, optical, acoustic, and thermal readouts offer distinct advantages that include miniaturization and low power [98]; high photostability and precise near-field amplification [105,107]; label-free, real-time kinetics [113]; and calorimetric sensitivity to binding energetics [123], respectively. Translational success, however, correlates less with the nominal platform than with alignment across recognition, interface, and measurement. Reagentless voltametric formats that incorporate internal standards or frequency-domain controls have proven resilient to drift [280,281] and optical designs that leverage plasmonic amplification while maintaining photostability sustain stable signal-to-noise at low abundance [105,107]. In short, platform choice determines how a signal is generated; stability and reproducibility follow from how the device is built and measured.

Evidence from clinically relevant matrices underscores these priorities. Differential-pulse voltammetric detection of SARS-CoV-2 spike protein at ~91 pM in human saliva and serum within ~35 min provides a concrete benchmark for time-to-result and matrix compatibility, achieved by aligning sequence properties, surface chemistry, and readout conditions [257]. At the same time, the literature documents why routine performance can diverge from headline LoDs. Studies show that biofouling and monolayer desorption are notable causes of signal degradation and that electrode microstructure and packing density confound absolute currents and inter-device comparisons [271,277,278]. Moreover, workflows that require exogenous mediators (e.g., faradaic EIS with ferri/ferrocyanide) or repeated calibrations can impede POC adoption due to drift and added steps [283,288]. Calibration-free voltametric strategies, notably frequency-domain interrogation, mitigate drift and have been validated even for continuous in vivo measurements [280,281]. Likewise, mediator-free impedance formats reduce steps and preserve single-step operation, though they require effective blockers and drift correction to withstand undiluted serum [258,294]. Viewed through REASSURED, designs that shorten hands-on steps, stabilize baselines, and report uncertainty transparently are those with the clearest route to deployment [206,207].

The trajectory that emerges from these findings is pragmatic and points to a practical synthesis for translation. First, select for switchability and matrix-tolerant motifs, assisted by ML and high-throughput screening, to raise the baseline quality of candidates entering device development. Second, immobilize for stability, using chemistries and architectures that preserve conformation, control density and orientation, and resist fouling in the specific biological fluid of interest. Third, transduce with calibration-light methods that include internal standards or frequency-domain controls so that small drifts and handling variability do not propagate into quantification error. Fourth, report performance in forms that predict routine use and not only LoD, but also dynamic range, limit of quantification, matrix-spike recovery, time-dependent drift, and inter/intra-assay variability, so that cross-study comparisons become meaningful guides for clinical translation. When these elements cohere, lateral-flow implementations gain sensitivity without sacrificing usability, and electrochemical formats benefit from more rapid, quantitative results in clinically representative samples.

A credible route to reproducible field performance is a closed-loop, computational–experimental workflow. In discovery, ML applied to round-resolved sequencing should prioritize sequences with features predictive of switching and matrix stability, while in design, structure-aware modeling can set probe orientation and surface density for the intended sample. Likewise, in fabrication and test, microfluidic screening and disciplined drift evaluation should shorten iteration cycles and as it pertains to operation, compact readers can apply embedded algorithms to stabilize signals under everyday handling. Within this loop, dual-aptamer and structure-switching architectures can be deployed where specificity or multiplexing is paramount, and mediator-free or calibration-light electrochemical protocols can be selected where workflow simplicity is decisive. In this configuration, machine learning and modeling function not as add-ons but as the backbone that targets the true bottlenecks; in particular, fouling, drift, and workflow, which ultimately determine whether aptamer biosensors move from compelling demonstrations to dependable tools.

## 6. Challenges and Future Directions

In addition to their promising applications, AI-driven strategies for aptamer discovery face several critical limitations that require careful consideration. A major concern lies in model interpretability, as many deep learning architectures function as “black boxes”, making it difficult to trace how specific sequence features influence predicted binding performance [306]. Dataset bias is another limitation as current training datasets are often limited in size and skewed toward certain targets which potentially restrict model generalizability. Overfitting remains a risk when complex models are trained on sparse or noisy experimental data, leading to strong in-sample performance but poor predictive accuracy for novel sequences [307]. Furthermore, the majority of AI-predicted aptamers still require wet-lab validation, and experimental confirmation lags behind computational prediction. This creates a gap between in silico models and real-world biosensor applications. Finally, the challenge of generalizing across diverse molecular targets persists, as models trained on one class of targets (e.g., proteins) may not readily transfer to small molecules or whole cells. Addressing these limitations will be crucial for building robust, reliable, and widely applicable AI-guided aptamer selection pipelines.

Aptamer-based biosensors are progressing from laboratory prototypes to clinical trials and commercialization. A few examples of aptasensors in clinical trials include the Tenofovir aptasensor (Trial ID: NCT04870671), continuous steroid monitoring in interstitial Fluid (Trial ID: NCT06980753), saliva-based COVID-19 test (Trial ID: NCT04974203) and pancreatic cancer stem cells detection (Trial ID: NCT05745415). Yet the number of commercialized aptasensor IVD products remain scarce. Key challenges to expanding the aptamer diagnostic commercialization include high initial R&D costs to verify the efficacy of a novel aptamer in terms of sensitivity and specificity, stiff competition with existing antibody-based diagnostics that necessitate clear justification of aptamer-based sensing benefits, inconsistency in analytical performance when transitioning to biosensing in human biofluids, response standardization for differentiation between positive and negative samples and prolonged clinical trial procedures to meet the strict regulatory guidelines for commercialization.

While AI and in silico aptamer selection can significantly speed up the screening of novel aptamers at scale, sensor compatibility and stability over time in complex human biofluids should be addressed to enable wider applications across various matrices such as whole blood, saliva, urine and sweat. Often observed in the literature is a drop in sensitivity when the sensors are used to detect spiked or native biomarkers in human samples. This can be partly attributed to biofouling and receptor surface degradation leading to signal drift overtime [258,271,294]. Thus, further investigations on signal normalization algorithm and development of alternative antifouling or surface passivation strategies are necessary to improve diagnostic reproducibility and accuracy, both are crucial parameters to fulfill in clinical trials for commercialization.

To conclude, the field of aptamer-based biosensing stands at an exciting juncture. Recent years have shown significant progress in integration of biophysical approaches including electrochemistry that could have significant advantages for point-of-care diagnosis. DNA nanotechnology also presents a variety of solutions for solving the challenges of sensitivity and specificity relating to nucleic acid biosensing. At the aptamer level, possibilities of integrations of chemistries beyond canonical DNA and RNA also allow a further approach to increase sensitivity and specificity. At the same time, there have been major breakthroughs in computational modeling that are starting to circumvent some of the bottlenecks around aptamer selection and optimization. Solving these challenges together leads to possibilities of multiplexed detection of panels of biomarkers, thereby widening the societal impact of aptamer-based biosensing for a variety of applications.

## Figures and Tables

**Table 3:** REASSURED criteria evaluation for POCT (modified from [206,207]).

Criteria	Description
Real-time connectivity	The tests are connected to a mobile reader to provide the diagnostic result
Ease of specimen collection	Specimen are collected by non-invasion sampling techniques
Affordable	The tests are cheap and affordable to the public
Sensitive	Avoid false negative
Specific	Avoid false positive
User-friendly	The tests are performed by the end users without difficulties
Rapid and Robust	The assays are repeatable and generate results within a short time
Equipment free	The tests are performed without the need of external instrument
Deliverable	The products should be accessible to all users

## Data Availability

No new data were created.

## Abbreviations

The following abbreviations are used in this manuscript:

3D	Three-Dimensional
AC	Alternating Current
aDNA	Alzheimer’s disease specific sequences
AI	Artificial Intelligence
ALP	alkaline phosphatase
AMBER	Assisted Model Building with Energy Refinement
ATP	Adenosine triphosphate
AuNPs	Gold Nanoparticles
AβO	Amyloid-β oligomers
BB	Bandrowski’s base
BiLSTM	Bidirectional Long Short-Term Memory
BLI	Biolayer Interferometry
BNP	B-type Natriuretic Peptide
BoNT/C	Botulinum neurotoxin type C
bp-SNA	bipolar silica nanochannel array
CA15-3	Carbohydrate antigen 15-3
*C_dl_*	Double Layer Capacitance
CG	Coarse-grained
CGM	Continuous Glucose Monitoring
CHA	Catalytic hairpin assembly
CNN	Convolutional Neural Network
CNTs	carbon nanotubes
CS/PtNPs	Chitosan-stabilized platinum nanoparticles
ctDNA	circulating tumor DNA
DAB	3,3′-diaminobenzidine
DL	Deep Learning
DPV	Differential Pulse Voltammetry
E-AB	Electrochemical Aptamer Biosensor
ECL	Electrochemiluminescence
EDC	1-ethyl-3-(3-dimethylaminopropyl) carbodiimide
EIS	Electrochemical Impedance Spectroscopy
ELISAs	Enzyme-linked immunosorbent assays
EVs	Extracellular vesicles
EXPAR	Exponential isothermal amplification reaction
FBS	Fetal Bovine Serum
Fc	Ferrocene
FET	Field Effect Transistor
FRET	Fluorescence resonance energy transfer
GOx	Glucose oxidase
GRNN	General Regression Neural Network
HCR	Hybridization chain reaction
HER2	Human Epidermal Growth Factor Receptor 2
HRP	Horseradish peroxidase
IL-6	Interlukin-6
ITO	Indium Tin Oxide
KDM	Kinetic Differential Measurement
KPC-2	*Klebsiella pneumoniae* carbapenemase 2
LAMP	Loop-mediated isothermal amplification
LbL	layer-by-layer
LFA	Lateral Flow Assay
LoD	Limit of Detection
LSTM	Long Short-Term Memory
LSPR	Localized surface plasmon resonance
MA-MN	Multivalent Aptamer Magnetic Nanozyme
MARQ-LFIA	Multiple Aptamer Recognition-based Quantum dot Lateral Flow Immunoassay
MB	Methylene blue
MCC	Matthews correlation coefficient
MCF-7	Michigan Cancer Foundation-7
MCTS	Monte Carlo Tree Search
MD	Molecular Dynamics
MEA	Multielectrode array
MFE	Minimum Free Energy
ML	Machine Learning
MM/PBSA	molecular mechanics Poisson–Boltzmann surface area
MOFs	Metal–organic frameworks
mRMR	Maximum Relevance Minimum Redundancy
MSS$A	Multiple secondary structure string alignment
MUC1	Mucin 1
NHS	N-hydroxysuccinimide
Ni-NTA	Ni-nitrilotriacetic acid
NLP	Natural Language Processing
NMR	Nuclear Magnetic Resonance
OECT	Organic Electrochemical Transistor
P(ANi-Lu)/GO/CS	Poly(aniline-luminol)/graphene oxide/chitosan
PD	Particle display
PD-L1	Programmed death-ligand 1
PfLDH	Plasmodium falciparum lactate dehydrogenase
PLIP	Protein–Ligand Interaction Profiler
POCT	Point-of-Care Testing
PPD	Paraphenylenediamine
PSA3	Prostate Cancer Antigen 3
QCM	quartz crystal microbalance
QDs	Quantum dots
RBMs	Restricted Boltzmann Machines
RCA	Rolling circle amplification
*R_ct_*	Charge Transfer Resistance
RF	Random forests
RMSD	Root-Mean-Square Distance
RNNs	Recurrent Neural Networks
RPA70A	Replication protein A 70 kDa subunit domain
SAMs	Self-assembled monolayers
SAW	surface acoustic wave
SELEX	Systematic Evolution of Ligands by Exponential Enrichment
SERS	Surface-enhanced Raman Spectroscopy
SPAAC	Strain-promoted azide-alkyne cycloaddition
SSAs	Structure-switching aptamers
SVM	Support Vector Machine
SWV	Square Wave Voltammetry
TFA	Thermal fluorescence analysis
TGF-β1	Transforming growth factor beta 1
Tₘ	Melting temperature
TMB	Tetramethylbenzidine
TNF-α	Tumor necrosis factor alpha
VAE	Variational Autoencoder
VEGF	Vascular Endothelial Growth Factor
VTM	Viral Transport Medium
